# Synergistic Organic–Inorganic Interface Engineering for Stable Zinc Metal Anodes in Aqueous Batteries

**DOI:** 10.1002/advs.75141

**Published:** 2026-04-20

**Authors:** Huaichong Sun, Yimin Chen, Jianwei Lu, Xiyuan Zhong, Zhihong Luo, Kaiwen Yang, Kun Luo, Aamir Shahzad, Muhammad Naveed Anjum, Weiwei Lei, Dan Liu, Aijing Ma

**Affiliations:** ^1^ Guangxi Key Laboratory of Optical and Electronic Materials and Devices Collaborative Innovation Center for Exploration of Nonferrous Metal Deposits and Efficient Utilization of Resources in Guangxi College of Materials Science and Engineering Guilin University of Technology Guilin Guangxi China; ^2^ Jiangsu Province Engineering Research Center of Intelligent Manufacturing Technology for the New Energy Vehicle Power Battery School of Materials and Engineering Changzhou University Changzhou China; ^3^ School of Science STEM College RMIT University Melbourne Victoria Australia; ^4^ Institute of Molecular Plus School of Chemical Engineering and Technology Tianjin University Tianjin China; ^5^ Department of Physics Government College University Faisalabad Pakistan; ^6^ State Key Laboratory of Advanced Separation Membrane Materials School of Chemical Engineering and Technology Tiangong University Tianjin China

**Keywords:** (002) plane, aqueous zinc metal batteries, dendrite, hydrogen‐bond network, solid electrolyte interphase (SEI)

## Abstract

In aqueous zinc‑ion batteries (AZIBs), uncontrolled dendrite growth and parasitic corrosion reactions critically limit long‑term stability. Constructing a robust organic–inorganic solid electrolyte interphase (SEI) has emerged as an effective strategy; however, the mechanistic origin of Zn‐anode stabilization remains insufficiently understood. Here, we in situ construct an ultrathin organic–inorganic hybrid SEI (Zn‐S‐RCOOH) on Zn using a multifunctional organic acid, mercaptosuccinic acid (MSA). The COOH‑rich organic outer layer restructures the interfacial hydrogen‑bond (HB) network, lowers H_2_O activity, and accelerates desolvation, whereas the inner ZnS layer provides fast Zn^2+^ migration pathways, collectively enhancing reaction kinetics and promoting (002) oriented Zn deposition. Owing to these synergistic effects, dendrite formation and corrosion are effectively inhibited. The MSA/Zn electrode operates stably for over 2400 h at 10 mA cm^−2^ and 5 mAh cm^−2^, and maintains>500 h stability even at a high depth of discharge (DOD, 81 %). Moreover, MSA/Zn||MnO_2_ full cells exhibited capacities of ∼189.7 and 142 mAh g^−1^ with high‐capacity retention (99.18 % and 97.55 %) at 0.3 and 1 A g^−1^, respectively. So, our findings proposed a rational interfacial‐engineering strategy for designing durable Zn metal anodes and advancing high‐performance aqueous zinc‐ion batteries.

## Graphical abstract

A multifunctional mercaptosuccinic acid enables an ultrathin organic–inorganic SEI (Zn‐S‐RCOOH) that synergistically regulates interfacial water and accelerates Zn^2+^ transport. The optimized hydrogen‐bond environment and ZnS‐guided deposition suppress dendrites, corrosion, and by‐product formation, delivering ultrastable Zn cycling and high‐performance MnO_2_ full‐cell operation.

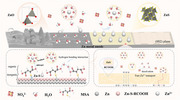

## 1 Introduction

Aqueous zinc‑ion batteries (AZIBs) have attracted significant attention due to their intrinsic safety, environmental friendliness, and low cost, positioning them as promising candidates for large‑scale energy storage [1, 2]. Metallic Zn further offers natural abundance, economic viability, a moderate redox potential (−0.76 V vs. SHE), and high theoretical gravimetric and volumetric capacities (820 mAh g^−1^ and 5855 mAh cm^−3^) [3, 4]. Despite these advantages, uncontrollable dendrite growth, the hydrogen evolution reaction (HER), and the accumulation of passivating by‑products significantly compromise the Coulombic efficiency (CE), cyclability, and overall lifespan of Zn anodes [5, 6]. These challenges arise primarily from inherent interfacial instability. The native solid electrolyte interphase (SEI), mainly composed of ZnO, exhibits poor ionic conductivity and high solubility in aqueous electrolytes [7, 8]. At the same time, random surface asperities induce non‑uniform electric‑field and ion‑concentration distributions [9]. Together, these effects promote uneven Zn deposition, leading to severe dendrite formation and corrosion.

To address these challenges, substantial efforts have focused on electrolyte engineering and surface modification to construct stable SEI layers on Zn anodes [10, 11]. Electrolyte‑derived organic or inorganic SEIs have attracted extensive attention [12]. However, SEI formed by spontaneous electrolyte decomposition is usually unstable [13]. Artificial SEIs, in contrast, can significantly improve rate capability and plating/stripping reversibility [14]. Although inorganic and polymer‑based SEIs fabricated via doctor‑blading offer certain interfacial benefits [15, 16], they typically suffer from non‑uniform thickness, limited ultrathin controllability, and insufficient adhesion to Zn [17]. In situ etching strategies overcome these limitations by producing ultrathin, conformal SEIs with strong chemical anchoring. However, inorganic acids such as HCl [18], HF [19], and H_3_PO_4_ [20] exhibit excessive reactivity toward Zn, generating inorganic crystalline SEIs that are vulnerable to H_2_O attack and often lead to severe corrosion and macroscopic pitting, especially under high DOD conditions [21]. Organic–inorganic hybrid SEIs derived from organic acids have shown greater reliability [22]. Yet, most studies emphasize their bulk properties while overlooking the distinct and potentially synergistic roles of their organic and inorganic domains [23, 24]. Notably, the organic component holds the potential to interact with water, leading to their confinement or repulsion, and prevents water from approaching and decomposing at the electrode surface, which is crucial for enhancing Zn metal anode stability. The inorganic component is essential for enhancing Zn^2+^ ion diffusion kinetics and suppressing dendrite formation. Despite this promise, the rational design and mechanistic understanding of such targeted hybrid SEIs remain underexplored.

In this study, we utilize a multifunctional organic acid, mercaptosuccinic acid (MSA), whose functional groups exhibit distinct reactivity toward Zn, making the rational construction of an organic–inorganic hybrid SEI. The highly nucleophilic ─SH group combines with Zn to construct an inorganic ZnS inner layer, whereas the less reactive ─COOH groups assemble into an organic outer layer. The COOH‑rich organic region reorganizes the interfacial hydrogen‑bond network, lowers H_2_O activity, and facilitates Zn^2+^ desolvation. Simultaneously, the nanoscale ZnS inner layer provides fast Zn^2^^+^ transport channels, while the SEI‑induced micro‑topographical regulation homogenizes electric‑field and ion‑concentration distributions, promoting uniform (002)‑oriented Zn deposition. Owing to the synergistic functions of the organic and inorganic components, dendrite growth and water‑induced corrosion are effectively suppressed, and the Zn‑anode reaction kinetics are markedly enhanced. Consequently, the MSA/Zn anode achieves exceptionally stable cycling for 2400 h at 10 mA cm^−2^ and 5 mAh cm^−2^, and sustains >500 h operation even under a high DOD of 81 % (20 mA cm^−2^, 20 mAh cm^−2^). Full cells based on MSA/Zn||MnO_2_ deliver high capacities of 189.7 and 142 mAh g^−1^ with excellent retention (99.18 % and 97.55 %) at 0.3 and 1 A g^−1^, respectively.

## 2 Results and Discussion

Efficient Zn^2+^ migration and effective mitigation of interfacial water activity are both essential for suppressing dendrite formation and corrosion; thus, a stabilization strategy must concurrently address ionic transport and water regulation. A rationally designed hybrid SEI can meet these requirements by utilizing its inorganic component to facilitate Zn^2+^ transport and its organic component to suppress corrosion. Thiol‐containing molecules are known to exhibit strong affinity toward metals, forming stable metal‐sulfur bonds such as Ag‐S [25], Au‐S [26], and Zn‐S [27]. Therefore, the ─SH groups in MSA are expected to react with Zn to produce an inorganic ZnS inner layer. Compared with ZnO, ZnS possesses intrinsically lower Zn^2+^ migration barriers [28], favoring faster ion transport. Meanwhile, the ─COOH groups, which show weaker reactivity toward Zn but strong affinity toward water, can restructure and weaken the interfacial hydrogen‑bond network, thereby confining H_2_O molecules and reducing their activity [29]. Motivated by these complementary functions, MSA containing both ─SH and ─COOH groups was selected to construct a targeted organic–inorganic hybrid SEI on the Zn anode.

**FIGURE 1:**
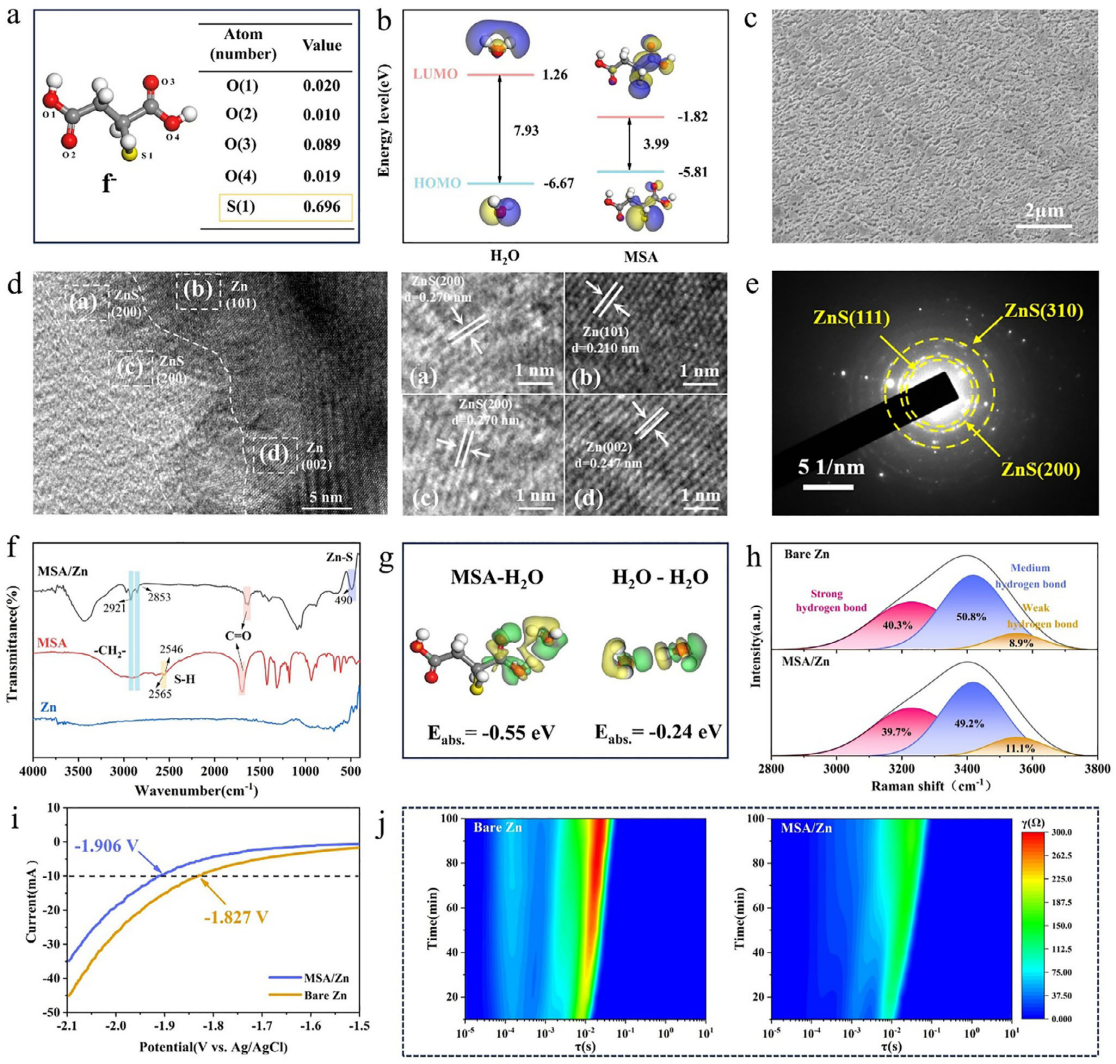
(a) The nucleophilic Fukui function in MSA, along with the marked possible sites and corresponding values. (b) LUMO and HOMO levels of H_2_O and MSA. (c) SEM images of MSA/Zn. (d) HRTEM (e) and SAED of MSA/Zn. (f) ATR‐FTIR spectra of bare Zn, MSA, and MSA/Zn. (g) The DFT calculation of the adsorption energy of MSA‐H_2_O and H_2_O‐H_2_O. (h) Raman spectra of bare Zn and MSA/Zn anode. (i) LSV curves of bare Zn and MSA/Zn anode. (j) DRT analysis of Zn||Zn symmetric cell by operando EIS evaluation during resting.

To verify the reactivity of ─SH and ─COOH groups with Zn, Fukui function calculations were performed to predict the reactive sites, which were labelled as O_1_, O_2_, O_3_, O_4_, and S1 (Figure 1a). According to the results, the f^−^ value represents the nucleophilic Fukui function. The S1 site shows the highest value (0.696), indicating that the ─SH group is the nucleophilic hotspot, more prone to electron acceptance and preferentially forming Zn─S bonds [30]. Furthermore, the highest occupied molecular orbitals (HOMO) energy levels and lowest unoccupied molecular orbitals (LUMO) energy levels were evaluated to evaluate the electron‐transfer capability of MSA and H_2_O at the Zn anode (Figure 1b). The calculated results reveal that compared with H_2_O (7.93 eV), MSA exhibits a smaller energy gap (3.99 eV). In addition, the LUMO energy level of MSA (−1.82 eV) is lower than that of H_2_O (1.26 eV), suggesting that MSA is more prone to electron acceptance and preferentially reacts with Zn foil to form SEI [31]. MSA treatment produces a smoother surface with uniform microtopography (Figure 1c); on the contrary, the bare Zn shows numerous protrusions, pits, and scratches (Figure S1). Cyclic voltammetry (CV) was used to evaluate the double‐layer capacitance (Figure S2). The double‐layer capacitance of MSA/Zn (261.12 µF cm^−2^) was significantly higher than that of bare Zn (83.96 µF cm^−2^), demonstrating a larger electrochemically active surface area (ECSA) for the MSA/Zn electrode, leading to more electroactive sites.

Multiple detection methods were used to determine the surface component of MSA/Zn. The XPS survey shown in Figure S3 indicates the existence of C, Zn, S, and O elements (Figure S3a). The fitted C 1s spectrum (Figure S3b) shows three peaks at 284.8, 285.8, and 288.7 eV, corresponding to the C─C, C─S, and O═C─O groups. The resolved Zn 2p signal (Figure S3c) displays two pairs of peaks at 1021.7 and 1044.8, 1022.8 and 1045.9 eV, corresponding to split peaks of Zn 2p_3/2_, Zn 2p_1/2_, and ZnS 2p_3/2_, ZnS 2p_1/2_. The resolved S 2p spectrum (Figure S3d) presents two peaks at 162.3 and 163.5 eV, corresponding to S 2p_3/2_ and S 2p_1/2_, which indicate that a binding interaction with the Zn surface is highly likely to have occurred. The high‐resolution O 1s signal (Figure S3e) presents a peak at 531.8 eV, assigned to the O═C─OH group.

The components of the SEI on MSA/Zn were further investigated by transmission electron microscope (TEM). The images and elemental mapping revealed good overlaps of Zn, S, and C elements, indicating a uniform distribution of components (Figure S4). In Figure 1d, well‐resolved lattice fringes were observed, which could be indexed to the ZnS (200) plane with an interplanar spacing (d) of 0.270 nm, as well as the Zn (101) plane (d = 0.210 nm) and Zn (002) plane (d = 0.247 nm). Selected area electron diffraction (SAED) patterns (Figure 1e) further proved the existence of ZnS and Zn due to the clear diffraction rings corresponding to ZnS (111), (200), and (310). Also, the average thickness of the SEI layer, measured from the high‐resolution TEM (HRTEM) micrograph, was determined to be approximately 8 nm (Figure 1d).

Attenuated total reflectance Fourier transform infrared (ATR‐FTIR) spectra shown in Figure 1f demonstrated two characteristic peaks at 2546 and 2565 cm^−1^ in the MSA spectrum, which were attributed to the stretching vibrations of S─H bonds. These peaks did not appear in the MSA/Zn spectrum. A characteristic peak of Zn─S bonds could be observed at 490 cm^−1^ in the MSA/Zn spectrum. ─SH groups in MSA reacted with Zn, which could be confirmed because of the formation of Zn─S bonds. At the same time, the peak assigned to C═O was observed, which is redshifted compared to MSA, possibly due to the intermolecular HB. In addition, two peaks at 2921 and 2853 cm^−1^, attributed to the stretching vibrations of methylene groups (─CH_2_─), were recorded in both the MSA and MSA/Zn spectra without peak shift, suggesting that the S‐RCOOH structure of MSA was preserved on the MSA/Zn surface.

Density functional theory (DFT) calculations were conducted to determine the adsorption energies of MSA‐H_2_O and H_2_O‐H_2_O (Figure 1g). The comparison of the adsorption energy of MSA‐H_2_O (−0.55 eV) and H_2_O‐H_2_O (−0.24 eV) indicates that the ─COOH group of MSA exhibits stronger interactions with H_2_O molecules, thereby altering the HB network among interfacial H_2_O molecules [32]. As shown in Figure 1h, the vibrations of O─H (2800–3800 cm^−1^) were divided into Gaussian components corresponding to three distinct HB environments: strong, medium, and weak HBs. The proportion of weak HBs at the MSA/Zn electrode interface (11.1 %) was higher than that at bare Zn (8.9 %), implying the weakened activity [33], thereby suppressing the hydrogen evolution reaction current, as evidenced by the linear sweep voltammetry (LSV) curves (Figure 1i).

To further analyze the corrosion behavior of the cell under resting conditions, the interfacial impedance of the electrode was monitored. The distribution of relaxation times (DRT) and electrochemical impedance spectroscopy (EIS) analyses (Figure 1j and S5) revealed that the interfacial impedance of MSA/Zn was significantly lower than that of bare Zn. During the resting period, the impedance of bare Zn increased markedly, whereas that of MSA/Zn increased only slightly (Figure S5). DRT analysis in Figure 1j showed that a peak at a relaxation time (τ) of 0.01 s can be observed for both bare Zn and MSA/Zn electrode. The bare Zn electrode exhibits a pronounced peak, whereas the MSA/Zn cell shows only a minor peak at the same τ value, indicating that substantial side products such as Zn_4_SO_4_(OH)_6_·5H_2_O were accumulated on bare Zn. From the Tafel plots (Figure S6), the corrosion current density of MSA/Zn (0.87 mA cm^−2^) was lower than that of bare Zn (1.88 mA cm^−2^). The morphology and structure of bare Zn and MSA/Zn during immersion were monitored. X‐ray diffraction (XRD) patterns (Figure S7) showed that new peaks assigned to Zn_4_SO_4_(OH)_6_·5H_2_O were observed after immersing the bare Zn anode in the electrolyte for 4 days, and the peak intensity increases significantly with the prolonged immersing time. On the contrary, the characteristic peak of Zn_4_SO_4_(OH)_6_·5H_2_O was not detected on MSA/Zn during this process. Scanning electron microscope (SEM) images further prove that the formation of large flakes on bare Zn (Figure S8), while for MSA/Zn, the surface remains as clean as the pristine stage. These results collectively confirm that the organic–inorganic SEI effectively suppressed hydrogen evolution and the formation of by‐products, thereby inhibiting corrosion reaction.

**FIGURE 2:**
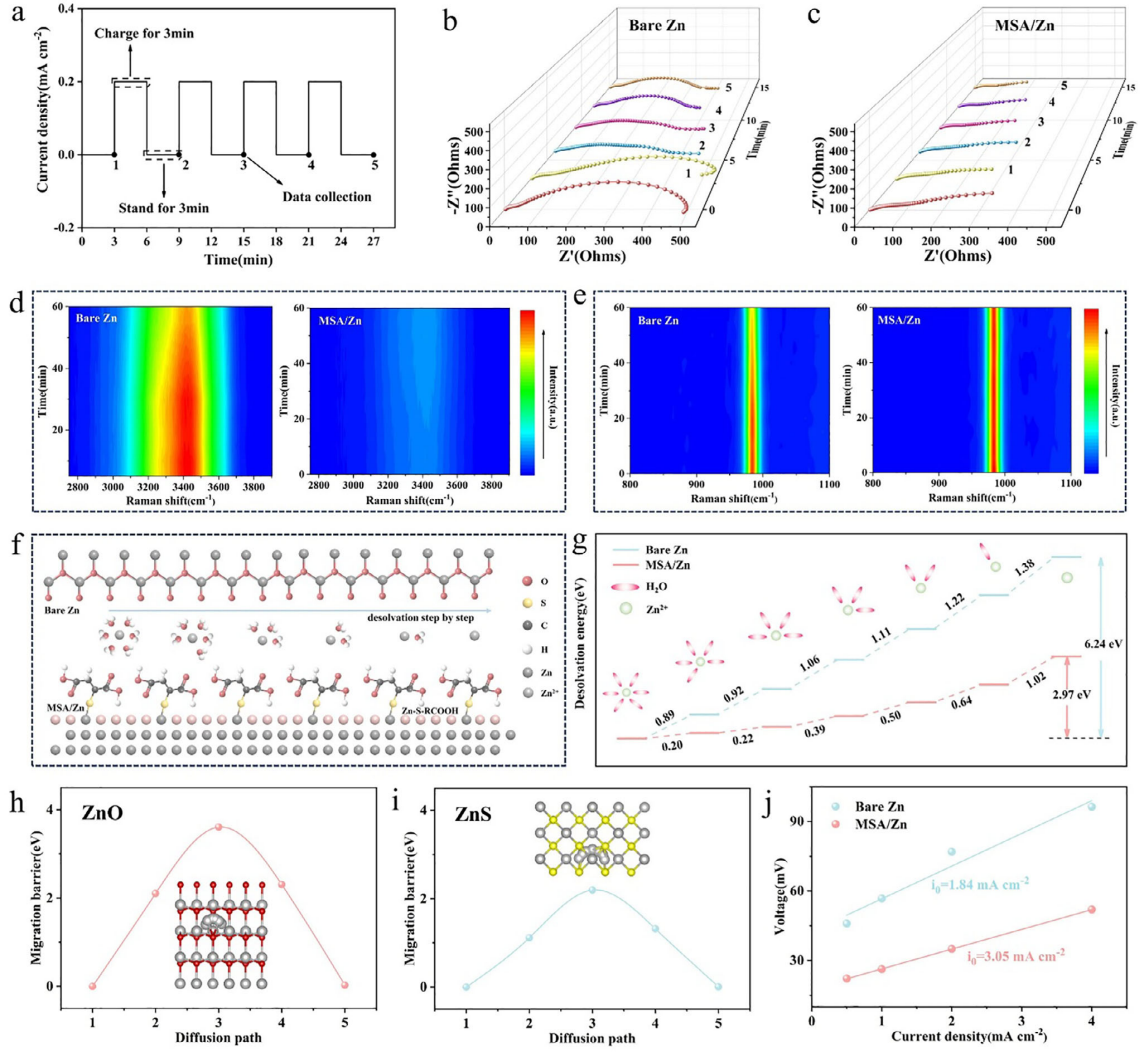
(a) Deposition processes in Zn||Zn symmetric cells. Plots of impedance change of symmetric cells with bare Zn (b) and MSA/Zn (c) anodes after charging and resting at a current density of 10 mA cm^−2^ for 3 min. (d,e) In situ Raman spectra recorded during the deposition process in Zn||Zn symmetric cells. (f) Schematic illustration of desolvation on bare Zn and MSA/Zn anodes. (g) The desolvation energy barrier of hydrated Zn^2+^ on bare Zn and MSA/Zn anodes. Zn^2+^ migration barrier diagram in (h) ZnO and (i) ZnS. (j) The exchange current density of bare Zn and MSA/Zn anodes.

To further investigate the electrode process, in situ characterization (EIS and Raman) and theoretical calculations were employed to elucidate the variation of interfacial resistance and ion concentration. In situ EIS was conducted to investigate the evolution of interfacial resistance during Zn deposition. As shown in Figure 2a, the Zn||Zn symmetric cell was continuously charged, followed by a 3 min rest period before EIS measurements were performed to collect impedance spectra at positions 1, 2, 3, 4, and 5. As illustrated in Figure 2b,c, the symmetric cell with bare Zn exhibited high and irregular interfacial resistance. In contrast, the symmetric cell with the MSA/Zn showed a smaller resistance than bare Zn, and tended to decrease slightly, confirming the stable electrode/electrolyte interphase.

Furthermore, in situ Raman spectroscopy was employed to probe the variations in HB intensity and SO_4_^2−^ concentration at the electrode/electrolyte interface during cycling. Figure 2d shows that the HB intensity at the MSA/Zn interface was markedly lower than that at bare Zn. This was mainly attributed to the organic component ─RCOOH groups, which anchored interfacial H_2_O molecules through HB, thereby reducing their activity [34]. As a result, hydrogen evolution and other parasitic reactions at the interface were suppressed, ensuring interfacial stability. Meanwhile, the Raman signal at 980 cm^−1^ corresponded to SO_4_^2−^, which was associated with Zn^2+^ concentration changes (Figure 2e). For bare Zn, the SO_4_^2−^ signal significantly decreased during Zn deposition, accompanied by a sharp decline in Zn^2+^ concentration, likely due to the formation of abundant by‐products. In contrast, the SO_4_^2−^ signal in MSA/Zn remained nearly unchanged, indicating negligible consumption of SO_4_^2−^ and consequently a stable Zn^2+^ concentration. The depletion of Zn^2+^ ions at the bare Zn interface induced concentration polarization, which triggered dendrite formation, whereas the stable Zn^2+^ concentration at the MSA/Zn interface contributed to the inhibition of dendrite growth [35].

Since the outer ─COOH‐rich organic part exhibits greater affinity to H_2_O, this may affect the desolvation process. To further validate this function, the desolvation processes at the interfaces of bare Zn and MSA/Zn anodes were simulated, and the corresponding energy barriers were calculated. Figure 2f, Figures S9 and S10 depict the stepwise removal of coordinated H_2_O molecules from the hydrated Zn^2+^ ion at both interfaces. As shown in Figure 2g, compared with the desolvation process at the bare Zn interface, the energy barrier for removing each coordinated H_2_O at the MSA/Zn interface is reduced. The energy (completely removed all six coordinated H_2_O molecules) was around 2.97 eV for MSA/Zn, notably lower than that of bare Zn (6.24 eV). This reduction implies that the interaction between the ─COOH groups and H_2_O molecules facilitated the detachment of coordinated H_2_O, thereby accelerating the desolvation process. The activation energy (E_a_), based on the charge transfer resistance (R_ct_) of the Arrhenius equation (Figure S11), further proved that the desolvation barrier of MSA/Zn was markedly reduced compared to bare Zn (41.73 kJ mol^−1^). These results demonstrate that the strong interactions between ─COOH groups and H_2_O molecules significantly promote the desolvation process.

Meanwhile, the effect of the inorganic component on Zn^2+^ ion migration was also investigated. Figure 2h,i presents the migration barrier of ZnO and ZnS, with the Zn^2+^ ions migration paths marked therein. The calculated results revealed that the migration barriers of Zn^2+^ ions in ZnS were significantly lower than those in ZnO, with each migration step in ZnS exhibiting a reduced energy barrier. This indicates that the inner inorganic ZnS part facilitates Zn^2+^ ion migration. In order to investigate the effect of COOH on Zn^2+^ migration, we established a simplified model and applied DFT to calculate the energy barrier for zinc ions transfer through a monolayer of ZnS and MSA‐ZnS. The corresponding Zn^2+^ migration path and calculated results are shown in Figures S12 and S13. The calculation results reveal that the Zn^2+^ ions migration energy barriers in the ZnS/Zn and MSA/Zn electrodes are 1.5 and 0.2 eV, respectively, which demonstrates that ─COOH groups can effectively lower the energy barrier for Zn^2+^ ions transport. The Bruce‐Vincent method was employed to measure the Zn^2+^ transference number (Figure S14), revealing that the Zn^2+^ transference number of the MSA/Zn electrode (0.58) is much higher than that of the bare Zn (0.17) and ZnS/Zn (0.37). The diffusion coefficient of MSA/Zn (1.38 × 10^−13^ cm^−2^ s^−1^) is also higher than that of bare Zn (9.37 × 10^−15^ cm^−2^ s^−1^) and ZnS/Zn (5.39 × 10^−15^ cm^−2^ s^−1^). Furthermore, the exchange current density (*i_0_*) of MSA/Zn (3.05 mA cm^−2^) is significantly higher than that of bare Zn (1.84 mA cm^−2^), indicating that the synergistic effect of the organic–inorganic SEI enhances the reaction kinetics of the MSA/Zn electrode (Figure 2j).

**FIGURE 3:**
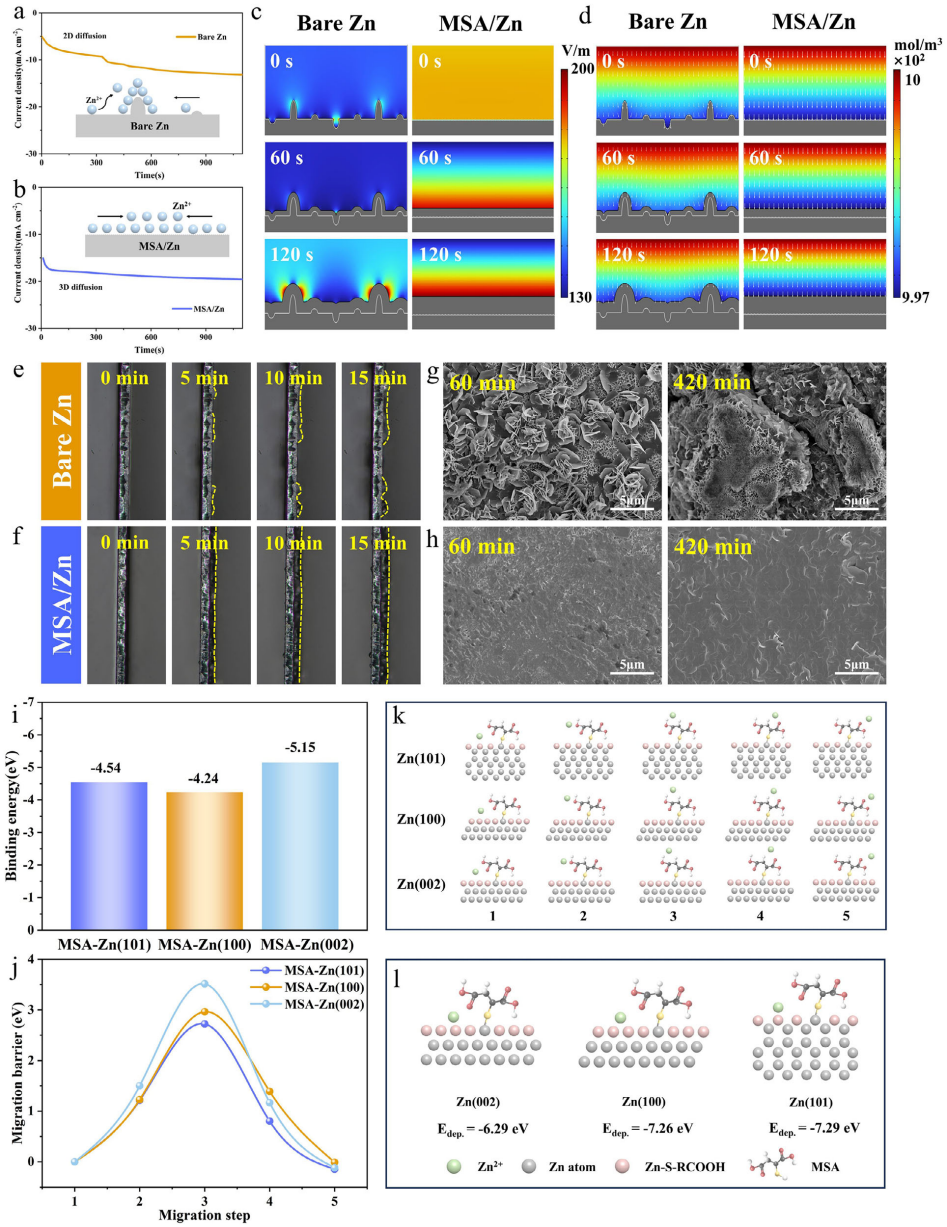
*I‐t* curves of (a) bare Zn and (b) MSA/Zn. COMSOL simulation of (c) the electric field and (d) Zn^2+^ concentration field distributions of bare Zn and MSA/Zn anodes during the deposition process. In situ optical microscopy observations in the (e) bare Zn and (f) MSA/Zn anodes at the current density of 10 mA cm^−2^. The surface morphology evolution of (g) bare Zn and (h) MSA/Zn anodes during the plating process at the current density of 1 mA cm^−2^. (i) The binding energy of MSA adsorbed on Zn planes. (j) Zn^2+^ migration barrier on Zn(101), Zn(100), and Zn(002) planes for MSA/Zn slabs. (k) Schematic illustration of Zn^2+^ horizontal migration step on Zn(101), Zn(100), and Zn(002) planes for MSA/Zn slabs. (l) Deposition energy of Zn^2+^ ion on (002), (100) and (101) planes of MSA/Zn.

The adsorption energy (Figure S15) of Zn^2+^‐MSA (−8.4 eV) is stronger than that of Zn^2+^‐H_2_O (−4.65 eV), implying that the organic–inorganic SEI possesses Zn affinity sites, which contribute to reducing the nucleation overpotential [36]. As shown in Figure S16, the nucleation overpotential of the MSA/Zn electrode (54.34 mV) is much lower than that of the bare Zn electrode (76.53 mV). Notably, the same trend was also observed in the CV curves (Figure S17). Additionally, it can be clearly seen from the curves that the electrochemically active surface area (A_e_) of MSA/Zn increased by 199.31 % compared with that of bare Zn, providing abundant active sites for Zn deposition and thus facilitating uniform deposition. When an overpotential of −150 mV was applied for deposition, as shown in Figure 3a, the bare Zn exhibits a continuous increase of current density, indicating that a 2D diffusion process is prolonged and intense, which results in non‐uniform deposition with the growth of dendrites. In contrast, for the MSA/Zn (Figure 3b), the initial Zn nucleation and 2D diffusion process occurred within 30 s, followed by 3D diffusion. Consequently, Zn^2+^ ions can be deposited uniformly and smoothly on the MSA/Zn electrode. COMSOL Multiphysics was used to simulate the deposition process by modelling the current density and Zn^2+^ concentration distribution. As shown in Figure 3c,d, due to the surface protrusions and pits, the local electric field enhancement and the non‐uniform Zn^2+^ concentration were observed on the bare Zn anode. As the deposition time prolonged, the dendrite formed and developed. On the contrary, the MSA/Zn with micro‐topography possess homogeneous distribution of electric field and Zn^2+^ concentration, resulting in uniform deposition during the prolonged time.

Morphology monitoring at both macro and micro scales confirmed the above conclusion. In situ optical microscopy was used to observe the surface morphology of different Zn electrodes in symmetric cells at a current density of 10 mA cm^−2^. Small protrusions began to appear on bare Zn after 5 min, and these protrusions grew larger and eventually formed undesirable Zn dendrites (Figure 3e). In contrast, the MSA/Zn surface maintained an ordered and dense structure after 15 min of deposition, with no obvious protrusions (Figure 3f). The micro‐morphology evolution of Zn anodes was investigated at different current densities (0.1, 1, and 4 mA cm^−2^. At the current density of 1 mA cm^−2^(Figure 3g), the formation of dendrites and by‐products was observed on bare Zn. The dendrites grew larger, and the amounts of by‐products increased with the enhancement in capacity. In contrast, during the deposition of MSA/Zn at 1 mA cm^−2^ (Figure 3h), the newly deposited Zn was observed to fill in the microtopography, after which the surface remained flat. Under different current densities and capacities, the MSA/Zn surface still maintained a dense and flat morphology, with no dendrites or corrosion products observed (Figures S18–S21). XRD patterns further revealed differences in Zn planes during Zn deposition (Figures S22–S24). For bare Zn, the (101) plane dominated, and the intensity ratio of I_(002)_/I_(101)_ decreased with increasing current density and capacity, accompanied by the appearance of diffraction peaks from byproducts. In contrast, Zn deposition on the MSA/Zn electrode was dominated by the (002) plane. The I_(002)_/I_(101)_ ratio continuously increased with current density and capacity, and no byproduct peaks were detected, even under harsh deposition conditions.

The relative texture coefficient (RTC) analysis was shown in Figure S25; the proportion of Zn(002) plane on bare Zn decreased from 26 % to 14 % with increasing deposition capacity, whereas that of the MSA/Zn electrode increased from 35 % to 74 %, indicating a progressive dominance of Zn(002)‐oriented deposition. To further elucidate the mechanism of Zn(002)‐preferred orientation, the binding energy between MSA and Zn planes is calculated as shown in Figure 3i. The binding energy between MSA and Zn(002) is much more negative than Zn(100) and Zn(101), demonstrating a stronger interaction with the (002) plane. Meanwhile, we calculated the Zn^2+^ migration barrier on different Zn planes for MSA/Zn slabs (Figure 3j), with the corresponding Zn^2+^ migration steps schematically illustrated in Figure 3k. The horizontal migration barrier of Zn^2+^ ion on the Zn(002), Zn(100), and Zn(101) planes is 3.51, 2.96, and 2.72 eV, respectively. This suggests that Zn^2+^ migration on the Zn(002) plane is significantly hindered. Furthermore, the deposition energy of the Zn^2+^ ion on different Zn planes was calculated (Figure 3l). The deposition energy of Zn^2+^ ion on the Zn(002), Zn(100), and Zn(101) planes is −6.29, −7.26, and −7.29 eV, respectively. The highest deposition energy demonstrates that the Zn^2+^ ion deposits on the Zn(002) plane preferentially with the lowest rate. According to Bravais’ law, the orientation of crystalline planes is determined by the growth rate of different planes. The faster‐growing planes tend to disappear gradually; therefore, the slowest‐growing (002) plane eventually becomes the dominant texture [37]. Moreover, the Zn(002) plane with the lowest surface energy exhibits reduced electrochemical activity toward hydrogen evolution and corrosion reactions. Thus, the increased proportion of Zn(002)‐oriented deposition promotes the stability of MSA/Zn further.

**FIGURE 4:**
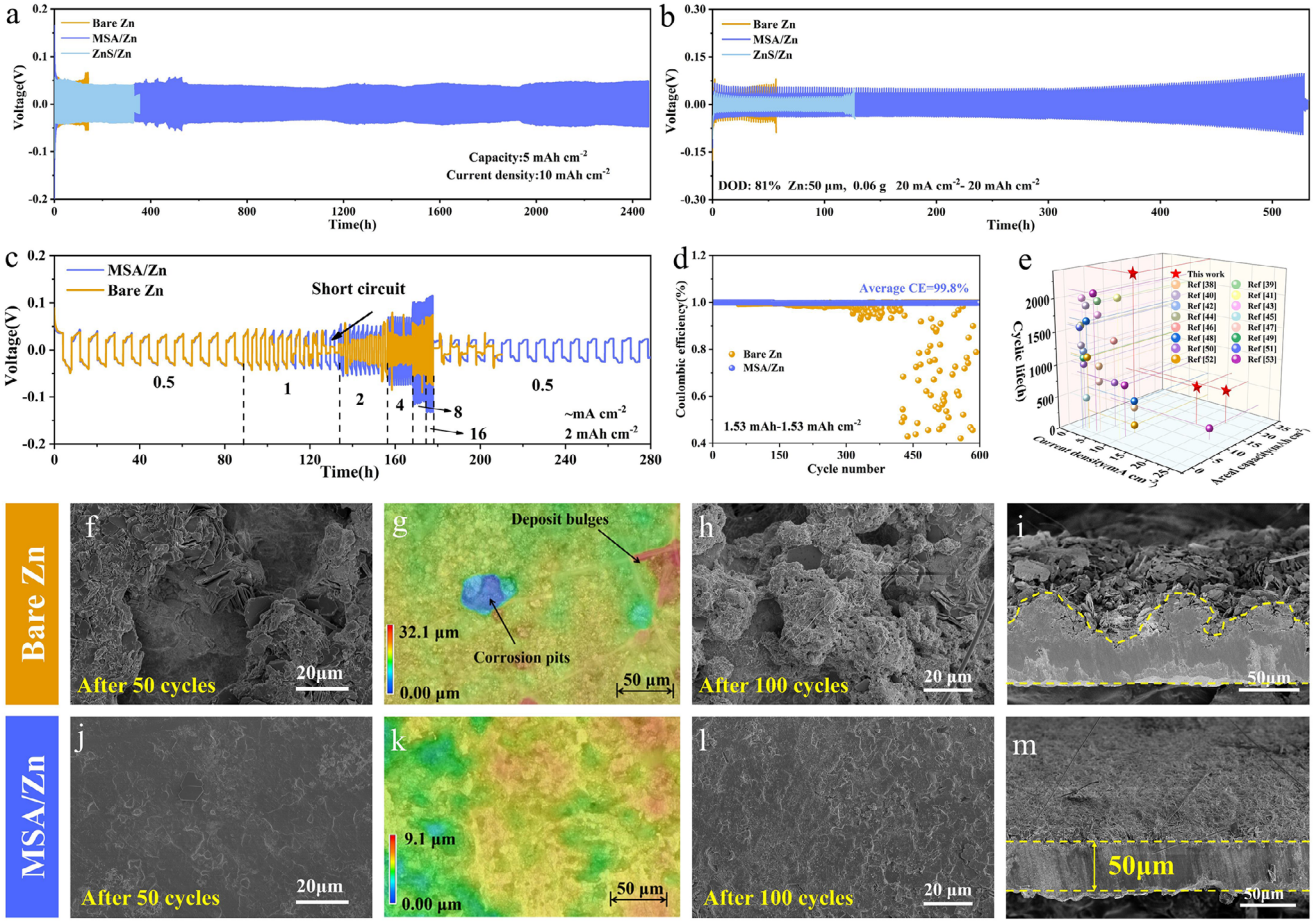
Cyclic performance of Zn||Zn cell with bare Zn, ZnS/Zn and MSA/Zn (a) at the current density of 10 mA cm^−2^ and capacity of 5 mAh cm^−2^, (b) at the current density of 20 mA cm^−2^ and capacity of 20 mAh cm^−2^, (c) rate capability of Zn||Zn cell at varied current density. (d) Coulombic efficiency (CE) of Zn||Cu cell with bare Zn and MSA/Zn at the current density of 1.53 mA cm^−2^ and capacity of 1.53 mAh cm^−2^. (e) Comparison of the cycling performance with that reported in recent works. Surface morphology and surface height variation on (f,g) bare Zn and (j,k) MSA/Zn electrodes after 50 cycles. Surface morphology and the cross‐section images of (h,i) bare Zn and (l,m) MSA/Zn after 100 cycles.

The electrochemical behavior of MSA/Zn was further evaluated in Zn||Zn symmetric cells and Zn||MnO_2_ full cells. As shown in Figure 4a, at a current density of 10 mA cm^−2^ and an aerial capacity of 5 mAh cm^−2^, the MSA/Zn electrode exhibited a markedly extended cycling lifetime of up to 2400 h, far exceeding that of bare Zn (142 h) and ZnS/Zn (325 h). Even under conditions of high depth of discharge, MSA/Zn demonstrated superior performance compared with bare Zn. For instance, at 65 % DOD (16 mA cm^−2^, 16 mAh cm^−2^), the symmetric cell with bare Zn failed within 70 h and exhibited a sharp increase in overpotential until short‐circuiting occurred, whereas MSA/Zn maintained stable cycling for nearly 600 h (Figure S26). At an even higher DOD of 81 % (20 mA cm^−2^, 20 mAh cm^−2^), MSA/Zn still sustained stable operation for more than 500 h (Figure 4b). For the ZnS/Zn electrode, the symmetric cell failed within 125 h; the cycling performance results further confirm that the presence of the organic component contributes to the long‐term stable operation of the Zn anode. The ability of Zn anodes to adapt to and recover from variations in current density is crucial for practical applications. Subsequently, symmetric cells were used sequentially at 0.5, 1, 2, 4, 8, and 16 mA cm^−2^ to test this property, followed by a return to 0.5 mA cm^−2^. As illustrated in Figure 4c, bare Zn failed to adapt well to current fluctuations. Finally, it was unable to recover its performance when the current density was reduced back to 0.5 mA cm^−2^. In comparison, MSA/Zn displayed excellent stability throughout the current density transitions, highlighting its robust interfacial properties.

Coulombic efficiency (CE), as a crucial parameter for assessing charge–discharge reversibility, was also evaluated at the current density of 1.53 mA cm^−2^ and capacity of 1.53 mAh cm^−2^. Unstable plating/stripping behavior directly leads to low CE and poor reversibility of Zn anodes. As shown in Figure S27, the Zn||Cu half‐cell using MSA/Zn exhibited a much narrower voltage gap (38.5 mV) than that of bare Zn (66.3 mV). The CE of the bare Zn cell (Figure 4d) dropped seriously after approximately 300 cycles, attributed to internal short‐circuiting induced by corrosion, hydrogen evolution, and interfacial side reactions. However, the MSA/Zn||Cu cell delivered a high and stable average CE of 99.8 % over 600 cycles, underscoring the promise of MSA/Zn for constructing highly durable Zn metal batteries. Compared with previously outstanding studies (Figure 4e; Table S1), the MSA/Zn exhibits equivalent or better performance [38, 39, 40, 41, 42, 43, 44, 45, 46, 47, 48, 49, 50, 51, 52, 53]. Morphology characterization of the electrodes after cycling provided further insights into the stable MSA/Zn. After 50 cycles at 10 mA cm^−2^ and 5 mAh cm^−2^, SEM images of bare Zn revealed severe surface degradation, with evident pits, protrusions, and large amounts of flake‐like by‐products (Figure 4f). In contrast, the MSA/Zn electrode remained smooth and compact after 50 cycles (Figure 4j). Optical profilometry offered a more direct visualization of surface roughness. The bare Zn electrode displayed large pits (blue regions) and protrusions (red regions) with a height variation of up to 32.1 µm (Figure 4g), whereas the MSA/Zn electrode maintained a much more uniform surface with a maximum height difference of only 9.1 µm (Figure 4k). And after 100 cycles, the bare Zn deteriorated further with full of dendrite and by‐products, while the compact and even surface of MSA/Zn morphology was largely preserved (Figure 4h,l). The cross‐section SEM images (Figure 4i,m) further revealed that the bare Zn electrode developed deep corrosion pits and porous protrusions, while the cycled MSA/Zn electrode preserved a flat morphology with nearly unchanged thickness (50 µm).

XRD patterns also revealed that these deposits are composed of by‐products. As the number of cycles increased, the I_(002)_/I_(101)_ ratio of the crystal planes for the MSA/Zn electrode remained at a relatively high level. In contrast, the I_(002)_/I_(101)_ ratio of the bare Zn electrode showed a decreasing trend, dropping to 0.53 after 100 cycles (Figure S28). Separators after 100 cycles were characterized (Figure S29), and from the SEM images and mapping results, the aggregates of dendrite and by‐products were clearly observed in the separator for bare Zn, further demonstrating the uncontrolled growth of dendrites and by‐products. In contrast, no deposits were detected in the separator for MSA/Zn. These observations further reflect the long‐life stability of the MSA/Zn electrode.

**FIGURE 5:**
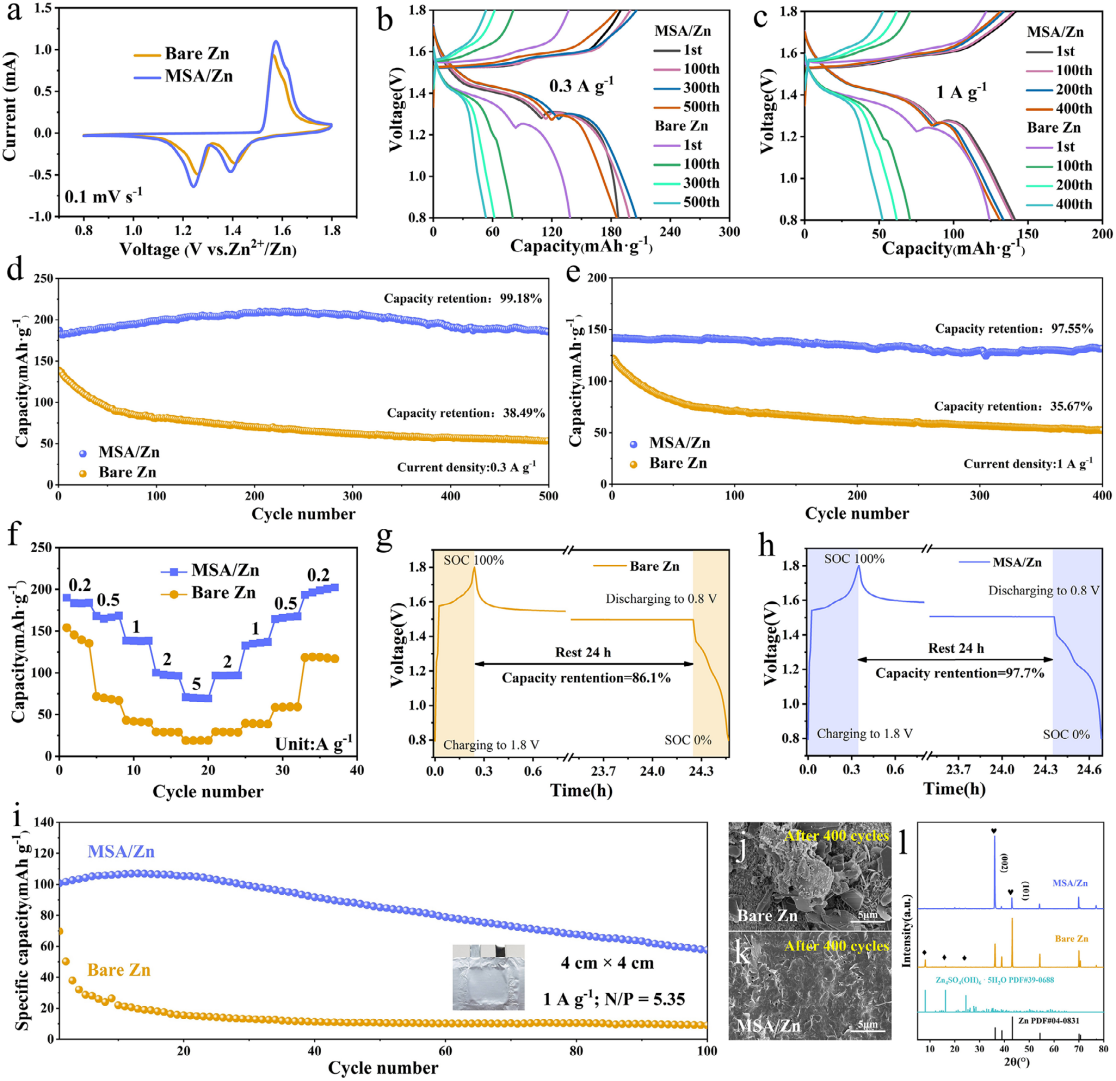
(a) CV curves, galvanostatic charge–discharge profiles (b) at 0.3 A g^−1^ (c) 1 A g^−1^, capacity retention (d) at 0.3 A g^−1^ (e) 1 A g^−1^, and (f) rate performance of Zn||MnO_2_ coin cell with bare Zn and MSA/Zn. Self‐discharge test of Zn||MnO_2_ full cell with (g) bare Zn and (h) MSA/Zn at 0.5 A g^−1^. (i) Capacity retention of Zn||MnO_2_ pouch cell with bare Zn and MSA/Zn. Surface morphology of bare Zn (j) and MSA/Zn (k) after 400 cycles. (l) XRD pattern of bare Zn and MSA/Zn.

To explore the modification effect of MSA/Zn in full cells and assess its practical potential, the electrochemical performance of Zn||MnO_2_ batteries was evaluated. The CV curves exhibit a pair of redox peaks assigned to the oxidation/reduction of Mn(IV) to Mn(III)/Mn(II) species (Figure 5a). Galvanostatic charge–discharge profiles of Zn||MnO_2_ and MSA/Zn||MnO_2_ full cells at the current density of 0.3 and 1 A g^−1^ are displayed in Figure 5b,c. At 0.3 A g^−1^, Zn||MnO_2_ delivered an initial capacity of 137.0 mAh g^−1^, which rapidly decayed during subsequent cycles. By contrast, MSA/Zn||MnO_2_ achieved a higher capacity of 189.7 mAh g^−1^ and exhibited much more stable long‐term cycling performance (Figure 5d). After 500 cycles, MSA/Zn||MnO_2_ retained 99.18 % of its capacity, while Zn||MnO_2_ retained only 38.49 %. A similar trend was observed at 1 A g^−1^ (Figure 5e), where the MSA/Zn||MnO_2_ cell delivered a capacity of 141.7 mAh g^−1^ with 97.55 % retention after 400 cycles, which was significantly higher than that of Zn||MnO_2_. The rate performance of both cells is shown in Figure 5f. The Zn||MnO_2_ cell suffered from a rapid capacity decline, and the capacity could not recover after returning to the initial current density. In contrast, MSA/Zn||MnO_2_ exhibited only a slight capacity loss at high rates and nearly full recovery when the current density was reduced, demonstrating excellent reversibility. The durability of the full cell was also evaluated by the self‐discharge test (Figure 5g,h). 24 h later, the MSA/Zn||MnO_2_ showed an impressive capacity retention of 97.8 %, better than that of Zn||MnO_2_ (86.1 %). The superior performance benefited from the excellent stability of the MSA/Zn anode.

After 400 cycles at 1 A g^−1^, SEM and EDS images of the Zn anodes revealed severe degradation for bare Zn (Figure 5j), with dense protrusions formed by deposited Zn and abundant by‐products accumulation, primarily in the form of Zn_4_SO_4_(OH)_6_·5H_2_O (Figures S30 and S31). In contrast, the MSA/Zn electrode surface remained relatively smooth (Figure 5k). XRD patterns of the cycled electrodes provided further insight (Figure 5l). Strong characteristic peaks of Zn_4_SO_4_(OH)_6_·5H_2_O were detected on bare Zn, consistent with the SEM observations of flake‐like by‐products. In contrast, no such peaks were observed for MSA/Zn, confirming the suppression of by‐product formation. Moreover, the I_(002)_/I_(101)_ ratio of bare Zn decreased significantly after cycling, while that of MSA/Zn increased, indicating that oriented deposition on the MSA/Zn electrode still maintains during the cycling process of the full cell. The 4 cm × 4 cm pouch cells with an N/P ratio of 5.35 were assembled, and the cyclic performance is shown in Figure 5i. With bare Zn, the pouch cell exhibits a capacity of 69.7 mAh g^−1^ at the first cycle, then the capacity drops sharply. For MSA/Zn, the pouch cell presents a capacity of about 100.7 mAh g^−1^ at the first cycle, and the capacity retention stabilized at 57.3 % after 100 cycles. These exceptional performances promote the construction of superior AZIBs.

## 3 Conclusion

In summary, a mercaptosuccinic acid (MSA) strategy was proposed, and a robust organic–inorganic hybrid SEI (Zn‐S‐RCOOH) on Zn metal was formed, in which the ─SH groups form a crystalline ZnS inner layer while the ─COOH groups modulate the interfacial water network, lower H_2_O activity, and facilitate Zn^2+^ desolvation. Consequently, this dual‑functional SEI architecture synergistically suppresses dendrite formation, mitigates parasitic reactions, and promotes uniform Zn deposition. In fact, the MSA/Zn anode delivers exceptional durability under stringent operating conditions, achieving 2400 h of cycling at 10 mA cm^−2^/5 mAh cm^−2^ and over 500 h of stable operation at 20 mA cm^−2^ with a high depth of discharge (81 %). When equipped as a full‑cell with MnO_2_ cathodes, improved capacity retention and practical applicability also appeared. This study not only constructs a rational strategy for designing targeted organic–inorganic SEIs but also provides valuable insights for advancing high‑performance Zn‑metal batteries.

## Author Contributions

Huaichong Sun, Yimin Chen, Jianwei Lu, Xiyuan Zhong: Conceptualization, Methodology, Investigation, Formal analysis, Data curation, Writing – original draft. Kaiwen Yang: Electrochemical measurements, Formal analysis. Aamir Shahzad, Muhammad Naveed Anjum: Electrochemical measurements, Calculation, Methodology, Investigation, Data Curation, Resources. Weiwei Lei, Dan Liu: Formal analysis, Software. Kun Luo, Zhihong Luo, Aijing Ma: Funding acquisition, Supervision, Project administration, Writing – review & editing.

## Conflicts of Interest

The authors declare no conflict of interest.

## Supporting information




**Supporting File**: advs75141‐sup‐0001‐SuppMat.docx.

## Data Availability

The data that support the findings of this study are available from the corresponding author upon reasonable request.
