# Supplementary material for: Synergistic Organic–Inorganic Interface Engineering for Stable Zinc Metal Anodes in Aqueous Batteries
Source: Adv Sci (Weinh). 2026 Apr 20;13(36):e75141. doi: 10.1002/advs.75141 (PMC13317556; doi:10.1002/advs.75141)
Supplement: Supplementary file 1 — Supporting File: advs75141‐sup‐0001‐SuppMat.docx. [file ADVS-13-e75141-s001.docx]

Supporting Information

**Synergistic Organic-Inorganic Interface Engineering for Stable Zinc Metal Anodes in Aqueous Batteries**

*Huaichong Sun, Yimin Chen, Jianwei Lu,* *Xiyuan Zhong*, *Zhihong Luo*, Kaiwen Yang, Kun Luo*, Aamir Shahzad, Muhammad Naveed Anjum, Weiwei Lei*, Dan Liu, Aijing Ma**

H. Sun, X. Zhong, Z. Luo,

Guangxi Key Laboratory of Optical and Electronic Materials and Devices, Collaborative Innovation Center for Exploration of Nonferrous Metal Deposits and Efficient Utilization of Resources in Guangxi, College of Materials Science and Engineering, Guilin University of Technology, Guilin, 541004, Guangxi, China

E-mail: luozhihong615@glut.edu.cn

J. Lu, W. Lei, D. Liu

School of Science, STEM College, RMIT University 124 La Trobe Street, Melbourne, VIC 3000, Australia

E-mail: weiwei.lei@rmit.edu.au

Y. Chen, K. Luo

Jiangsu Province Engineering Research Center of Intelligent Manufacturing Technology for the New Energy Vehicle Power Battery, School of Materials and Engineering, Changzhou University, Changzhou 213164, China

E-mail: luokun@cczu.edu.cn

A. Shahzad, M. N. Anjum

Department of Physics, Government College University, Faisalabad Allama Iqbal Road, Faisalabad, Pakistan

K. Yang

Institute of Molecular Plus, School of Chemical Engineering and Technology, Tianjin University, Tianjin, 300072 China

A. Ma

State Key Laboratory of Advanced Separation Membrane Materials, School of Chemical Engineering and Technology, Tiangong University, Tianjin 300387, China. E-mail: maaijing@tiangong.edu.cn

**Preparation of Bare Zn, ZnS/Zn, and MSA/Zn anodes**

Commercial Zn foil was cut into a Zn plate (0.05 mm thick, 16 mm diameter and weighing 0.06 g) to prepare bare Zn anodes. ZnS and PVDF were uniformly mixed in N-Methyl-2-pyrrolidone (NMP) at a mass ratio of 9:1, and the resulting slurry was coated onto the surface of Zn foil. The as-treated Zn foil was dried in a vacuum oven at 80°C for 12 h and then cut into Zn sheets for further use. Mercaptosuccinic acid (MSA) was dissolved in deionized water to prepare a solution with a concentration of 10 mmol·L^-1^. Zinc plates were immersed in the prepared MSA solution for 2 hours. Subsequently, the zinc plates were taken out, rinsed with deionized water, and then placed in a drying oven at 60℃ for drying, after which they were reserved for subsequent use.

**Preparation for MnO_2_ cathode**

A 0.08 mol L^-1^ MnSO_4_ solution was added to a 0.21 mol L^-1^ KMnO_4_ solution drop by drop. After the mixture was stirred for 30 min, it was transferred into a 100 mL Teflon-lined autoclave. Subsequently, the autoclave was placed in an oven and maintained at 160°C for 12 hours. Once the system cooled down to room temperature, the solid product was isolated via centrifugation with deionized water. The solid product was then placed in an oven at 60°C for approximately 24 hours to obtain MnO_2_. The prepared MnO_2_ was mixed with carbon black and PVDF at a weight ratio of 7:2:1 to form an ink. This ink was coated on the surface of a stainless-steel mesh and dried at 60°C for 12 hours, finally yielding the MnO_2_ cathode.

**Batteries assembling**

Bare Zn, MSA/Zn, Cu sheets (16 mm diameter), SS sheet (15.8 mm diameter), and MnO_2_ cathode (14 mm diameter) were used as electrodes. 2 M ZnSO_4_ was used as electrolyte, glass fibre (Whatman GF/D, 18 mm diameter)) was applied as a separator. All the batteries were assembled with CR2032 coin cells as follows: Zn||Zn symmetric cells were constructed using bare Zn and MSA/Zn as electrodes; Zn||Cu half-cells and Zn||SS half-cells were assembled with bare Zn and MSA/Zn serving as the anodes, and Cu sheets and SS sheets acting as the cathodes, respectively; Zn||MnO_2_ full cells were fabricated using bare Zn and MSA/Zn as the anodes and MnO_2_ as the cathode. In addition, the Zn||MnO_2_ pouch cell was fabricated using bare Zn and MSA/Zn as anode (0.02 mm thick, 4 cm × 4 cm, 200.0 mg weight) and MnO_2_ as cathode 4 cm × 4 cm, 99.6 mass loading).

**Batteries testing**

The battery performance was recorded on a standard battery testing system (CT-3008W-5V10mA, Neware Technology Limited). The deposition process was measured via galvanostatic charge/discharge using a Zn||Zn symmetric cell, with the following testing conditions: a current density of 0.1 mA cm^-2^ for 5 hours, a current density of 1 mA cm^-2^ for 7 hours, and a current density of 4 mA cm^-2^ for 2 hours. Coulombic efficiency was evaluated using Zn||Cu half-cells. The test protocol involved initial plating at 4 mA cm^-2^ for 1 hour, followed by cycling at a current density of 0.5 mA cm^-2^ and a capacity of 0.5 mAh cm^-2^. The cyclic and rate performance of Zn||MnO_2_ full cells were assessed at the current density of 0.1, 0.2, 0.3, 0.5, 1, 2, and 5 A g^-1^ within a potential window of 0.8 V to 1.8 V.

The electrochemical measurement performance was conducted in an electrochemical workstation (Squidstat Plus, 1.1). Tafel, EIS, nucleation overpotential, and chronoamperometry (CA) tests were performed on Zn||Zn symmetric cells assembled with bare Zn and MSA/Zn. LSV curves were recorded using a three-electrode system in a pure NaSO_4_ electrolyte, with Zn foil as the working electrode, Ag/AgCl electrode as the reference electrode, and Pt foil as the counter electrode, at a scanning speed of 5 mV s^−1^. CV tests on Zn||Zn symmetric cells and Zn||SS half-cells, assembled with bare Zn and MSA/Zn. Zn||Zn symmetric cells were assembled to obtain CV curves at different scan rates. The double-layer capacitance(*C*) was derived from the slope of the linear fit between scan rates(*v*) and capacitive currents (*i_c_*), as the following formula:^[1]^

$$i_{c}=Cv$$

where *i_c_* is calculated as half the difference between the currents at 0 V during positive/negative sweeps, *C* (μF cm^-2^) is double-layer capacitance, and *v* (mV s^-1^) is the scan rate.

The electroactive surface area (*A_e_*) of the Zn anode was estimated from the peak current (*i_p_*) in the CV curve using the following formula:^[2]^

$$i_{p}=3.67\times{10}^{5}n^{\frac{3}{2}}A_{e}cD^{\frac{1}{2}}\nu^{\frac{1}{2}}$$

where *i_p_* (mA) is the peak current, *n* (2) is the number of electrons, *A_e_* (cm^2^) is the electroactive surface area, *c* (2×10^-3^ mol cm^-3^) is the bulk concentration of Zn^2+^ ions, *D* (1×10^-10^ cm^2^ s^-1^) is the diffusion coefficient of Zn^2+^ ions, and *ν* (V s^-1^) is the scan rate.

Zn||Zn symmetric cells with bare Zn and MSA/Zn were assembled in order to measure Zn^2+^ ion transference ($t_{{Zn}^{2+}}$) number by using Bruce-Vincent method, the I-t curves were recorded with a bias voltage of 10 mV, the EIS plots were tested before and after polarization. The $t_{{Zn}^{2+}}$is calculated by the following formula:^[3]^

$$t_{{Zn}^{2+}}=\frac{I_{ss}(V-I_{0}R_{0})}{I_{0}(V-I_{ss}R_{ss})}$$

where *V* (10 mV) is the applied bias voltage, *I_ss_* (mA) and *R_ss_* (Ω) are the steady state current and resistance, respectively, and *I_0_* (mA) and *R_0_* (Ω) are the initial current and resistance.

Zn||Zn symmetric cells with bare Zn and MSA/Zn were assembled for electrochemical impedance measurements. The slope of the real part (*Z'*) of the low-frequency diffusion region in the electrochemical impedance spectroscopy against the reciprocal of the square root of the corresponding angular frequency (*ω*) was obtained and substituted into the following formula:^[4]^

$$D=0.5{(\frac{RT}{An^{2}F^{2}\sigma_{\omega}C})}^{2}$$

where *D* represents the ion diffusion coefficient (cm^2^ s^-1^); *R* is the gas constant ( J (K mol)^-1^); *T* is the temperature (K); *A* is the electrode surface area (cm^2^); *F* is the Faraday constant (C mol^-1^); *C* is the molar concentration of Zn^2+^ in the electrolyte (mol cm^-3^); *σ_ω_* is the Warburg factor, whose calculation formula can be referred to formula:

$$\sigma_{\omega}=\frac{Z'}{\omega^{-0.5}}$$

Where *σ_ω_* is the Warburg factor; *Z'* denotes the real part value of the low-frequency diffusion region, and *ω* represents the angular frequency value of the low-frequency diffusion region.

Activation energy (*E_a_*) was determined from the results of EIS conducted on symmetrical Zn||Zn cells with bare Zn and MSA/Zn. *E_a_* is calculated from the Arrhenius equation:^[5]^

$$\frac{1}{R_{ct}}=Aexp(-\frac{E_{a}}{RT})$$

where *R_ct_* (Ω) is the charge transfer resistance, *A* is the pre-exponential factor, *E_a_* (kJ mol^-1^) is the activation energy, *R* (J K^-1^ mol^-1^) is the gas constant, and *T* (K) is the Kelvin temperature.

The exchange current density for electrodeposition is analyzed according to the following equation:^[6]^

$$i\approx i_{0}\frac{F}{RT}\frac{n}{2}$$

where 𝜂 (mV) denotes the total overpotential, 𝑖 (mA cm^-2^) represents the current density, and 𝑖_0_ (mA cm^-2^) represents the exchange current density.

The relative texture coefficients (*RTCs*) of each Zn lattice plane of Zn anode are calculated according to the following formula:^[7]^

$${RTC}_{(hkl)}=\frac{I_{(hkl)}/I_{0(hkl)}}{Ʃ{(I}_{\left( hkl \right)}/I_{0\left( hkl \right)})}\times100$$

Where *I*_0(_*_hkl_*_)_ is the intensity of the standard oriented sample, *I*_(_*_hkl_*_)_ is the intensity obtained from the textured sample.

The DOD of Zn anode was calculated using the following equation:^[2]^

$$DOD=\frac{It}{\mathrm{mM}}\times100\%$$

where *I* (mA) is the applied current density, *t* (h) is the discharge time, *m* (g) is the mass of the Zn anode, and *M* (mAh g^−1^) is the theoretical specific capacity of the Zn anode (820 mAh g^−1^).

**Materials characterizations**

The surface morphology and related energy dispersive spectroscopy (EDS) were recorded with a scanning electron microscope (SEM, S4800, Hitachi). High-resolution transmission electron microscopy images (HRTEM), elemental mapping, and selected-area electron diffraction (SAED) patterns were collected using a transmission electron microscope (TEM, JEM-2100F, JEOL). FTIR and Raman were measured on a Fourier transform infrared (FTIR, IRtracer-100) and Raman spectrometer (Raman, Fisher DXR, Thermo Electron, American). In-situ Raman spectra were tested on a Raman spectrometer (Thermofisher, DXR2 ) with a home-made cell at a current density of 0.1 mA cm^-2^ and a capacity of 0.1 mAh cm^-2^, and data were recorded every 5 mins for 2 min. The optical observation of the deposition process was conducted on a dendrite observation microscope system (YUESCOPE YM710TR-Z). The texture analysis was conducted with an X-ray diffractometer (XRD, X’ Pert Pro, PANalytical B.V.) with a Cu-Kα target under a voltage of 40 KV.

**Density functional theory (DFT) calculation method**

The DFT calculation was performed by the Vienna Ab-initio Simulation Package (VASP), and the exchange-correlation energy was approximately described by the Perdew-Burke-Ernzerhof (PBE) functional based on the generalized gradient approximation (GGA). In all calculations, a cut-off energy with a value of 482 eV was used for the plane wave basis, and the convergence criteria for the ionic relaxation and 4 electronic self-consistent calculations were set to 0.02 eV Å^-1^ and 10^-4^ eV, respectively. Then the charge density difference was analyzed.

The migration energy barrier was calculated using a Cambridge sequential total energy package (CASTEP) in MS 2020. The exchange function was treated with the generalized gradient approximation (GGA) of Perdew-Burke-Ernzerhof (PBE) function. The DFT-D method was employed to calculate the van der Waals (vdW) interaction. Energy and force will not reach convergence until lower than 1×10^–5^ eV and 0.02 eV/Å, respectively. The Brillouin zones of the supercells were sampled by a 2 × 2 × 1 uniform k-point mesh, and a plane-wave basis set with an energy cut-off of 490 eV was employed. To avoid interactions between periodic images, a vacuum space of more than 15 Å was applied to all calculations. The desolvation energy was calculated like binding energy.

The adsorption energy was defined as the following equation:

$$\text{E}_{\text{ads}}\text{=}\text{E}_{\text{(*adsorbent)}}\text{ - }\text{E}_{\text{(*)}}\text{ -}\text{E}_{\text{(adsorbent)}}\text{ }$$

where $\text{E}_{\text{(*adsorbent)}}$ $\text{E}_{\text{(*)}}$and $\text{E}_{\text{(adsorbent)}}$represent the total energy of * adsorbent, * and adsorbent molecule, respectively.

The binding energy is defined as follows:

$$\text{E}\text{=}\text{E}_{\text{a-b}}\text{-}\text{E}_{\text{a}}\text{-}\text{E}_{\text{b}}$$

where $\text{E}_{\text{a-b}}$ is the total energy after binding, $\text{E}_{\text{a}}$ and $\text{E}_{\text{b}}$ are the energies of optimized molecules, respectively.

The exchange-correlation effects were treated with the generalized gradient approximation (GGA) employing the Perdew-Burke-Ernzerhof (PBE)functional. To characterize the chemical reactivity of the system, the Fukui function analysis was implemented based on Hirshfeld charge partitioning. Specifically, three functions of the Fukui function, nucleophilic (***f ^+^***), electrophilic (***f ^-^***), were calculated using the following formalism:

Nucleophilic Fukui function **(*f ^+^*)**:${\text{f}\text{ }}^{\text{+}}\left( \text{r} \right)\text{= }\text{ρ}_{\text{N}\text{+}\text{1}}\text{®}\text{ }\text{-}\text{ }\text{ρ}_{\text{N}}\text{®}$

Electrophilic Fukui function **(*f ^-^*)**: $\text{f }^{\text{-}}\left( \text{r} \right)\text{= }\text{ρ}_{\text{N}}\text{®}\text{-}\text{ρ}_{\text{N-1}}\text{®}$

where $\text{ρ}_{\text{N}}\text{®}$ denotes the electron population of atom k in original surface, $\text{ρ}_{\text{N}\text{+}\text{1}}\text{®}$ corresponds to the electron population in case after adding one electron to the cluster, and $\text{ρ}_{\text{N-1}}\text{®}$ represents the electron population in case upon removing one electron from the cluster.

**Comsol Simulation**

To simulate the distributions of the electric field and Zn^2+^ concentration field at the anode during the electrodeposition process, a 2D model was established and simulated based on COMSOL Multiphysics. In this model, the electrodes were set as boundary conditions with no thickness. The length of the Zn electrodes was set to 9 μm, with 4 μm between the anode and the cathode. The surface morphologies of bare Zn and MSA/Zn were constructed based on SEM observations. Specifically, the cavities on the MSA/Zn surface exhibited a depth of 0.5 μm and an inter-cavity spacing of 0.1 μm. To simulate the electrodeposition process, a current density of 5 mA cm^-2^ was applied to the cathode, while the anode potential was fixed at 0 V. The operating temperature was maintained at 298 K, and the initial concentration of the ZnSO_4_ electrolyte was set to 2 M. The simulation used the Butler-Volmer expression to describe the reaction kinetics of all electrodes, and the flux of each ion was calculated by the Nernst-Planck formulation.

**Supplementary Figures**

**
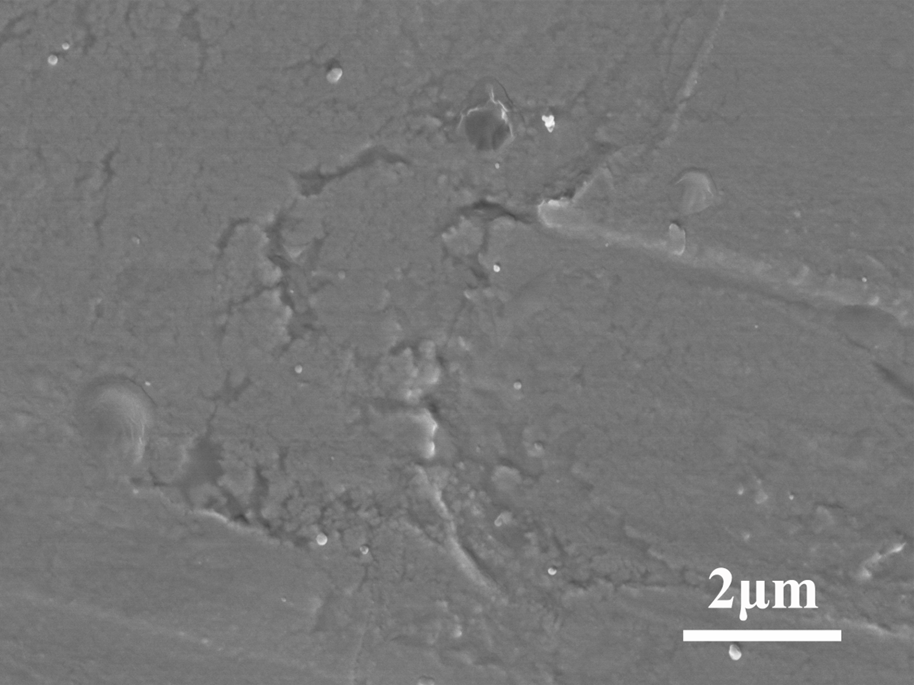
**

**Figure S1.** SEM images of bare Zn.


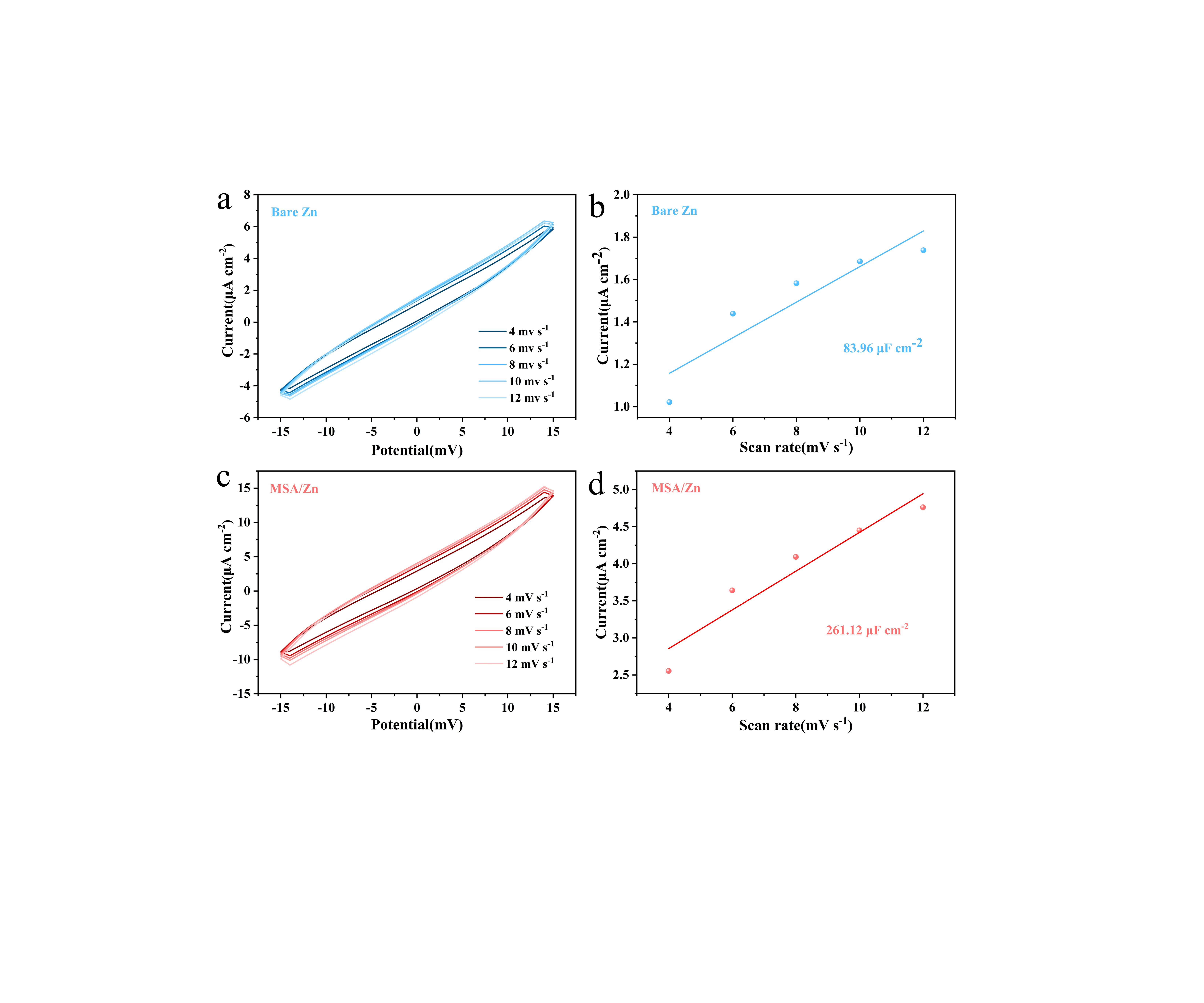


**Figure S2.** Voltage profiles of (a, b) bare Zn and (c, d) MSA/Zn symmetric cells under various scan rates from 4 to 12 mV s^−1^ and corresponding fitting results.

**
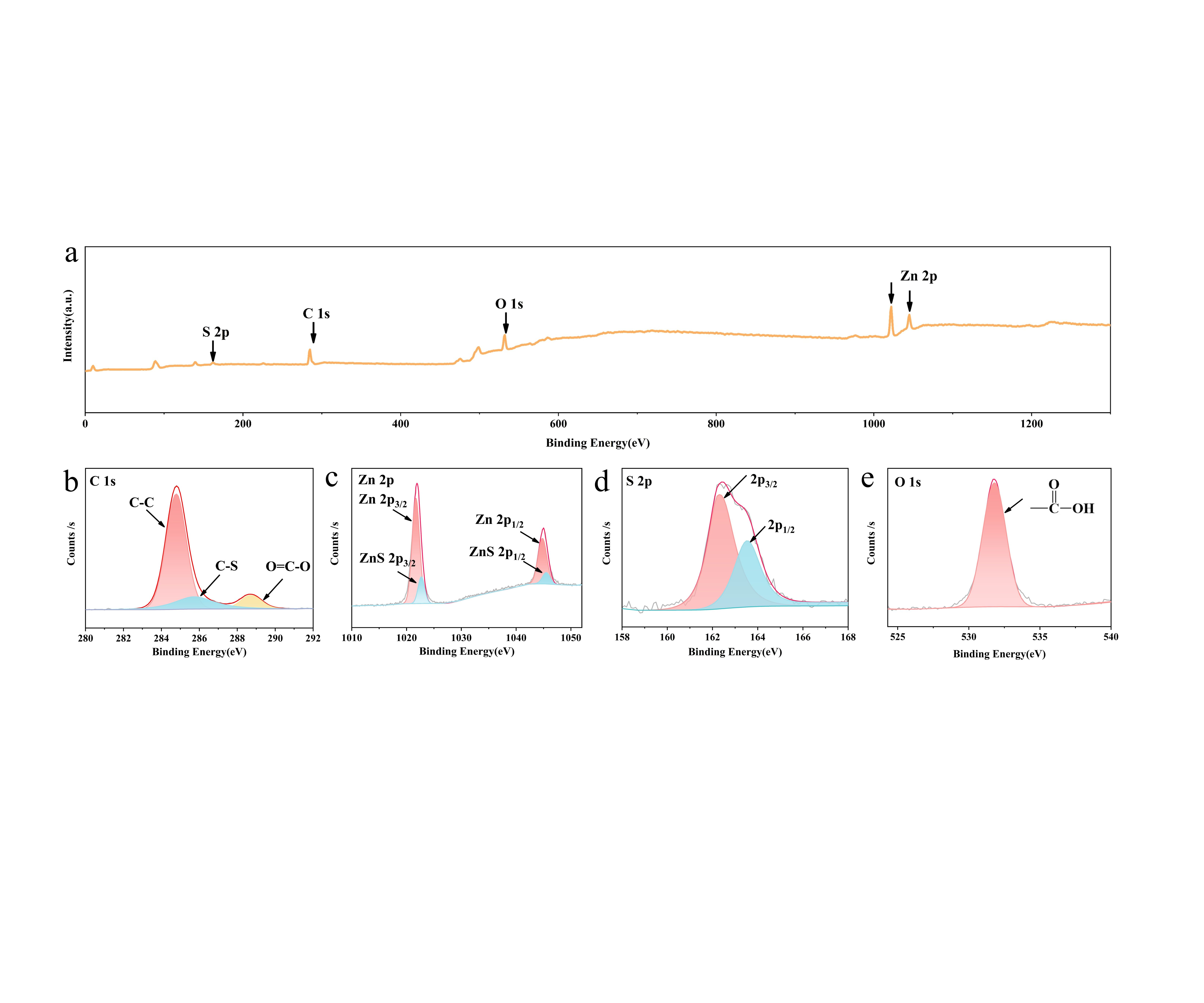
**

**Figure S3.** XPS survey of MSA/Zn (a), C 1s (b), Zn 2p (c), C 1s (d), and O 1s (e)spectra of MSA/Zn.


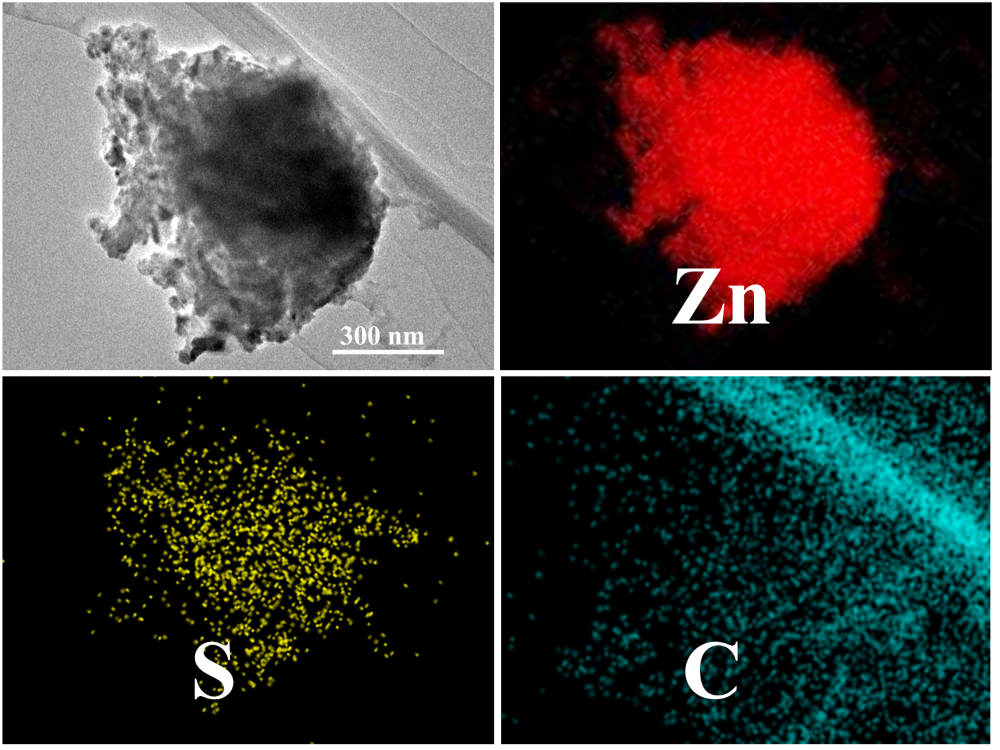


**Figure S4.** TEM image and Zn, S, O elemental mappings.

**
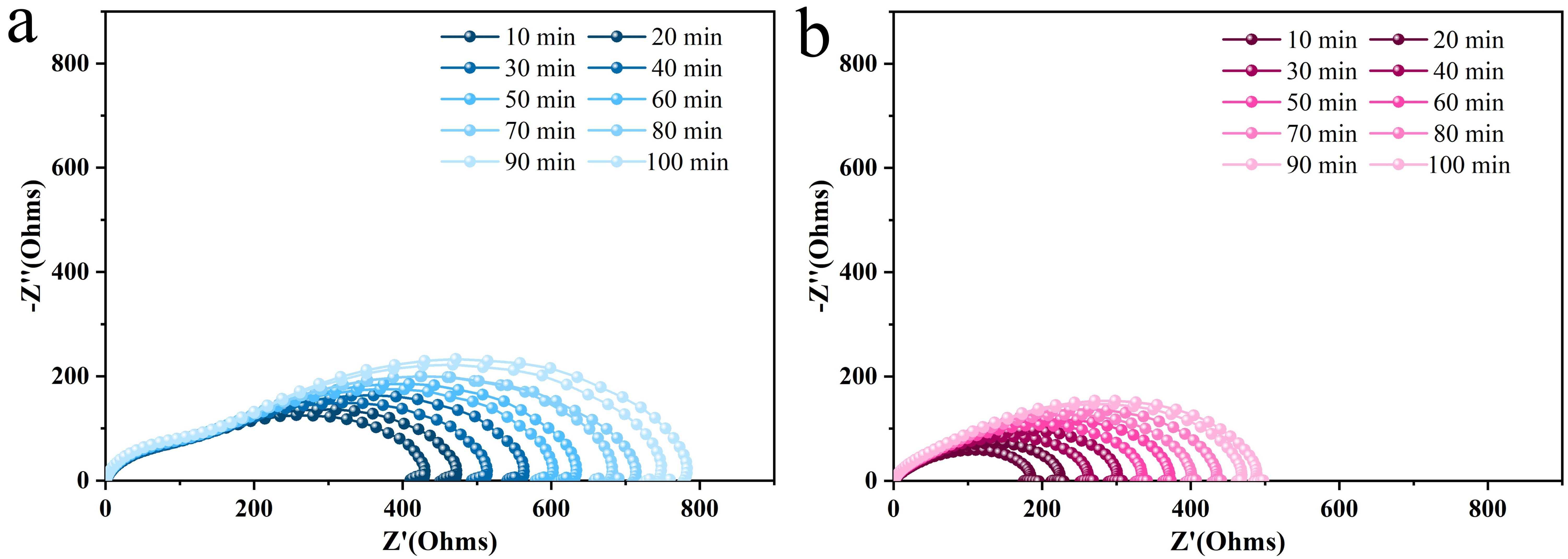
**

**Figure S5.** The EIS plots of Zn||Zn cell with bare Zn (a) and MSA/Zn (b) during continuous resting.

**
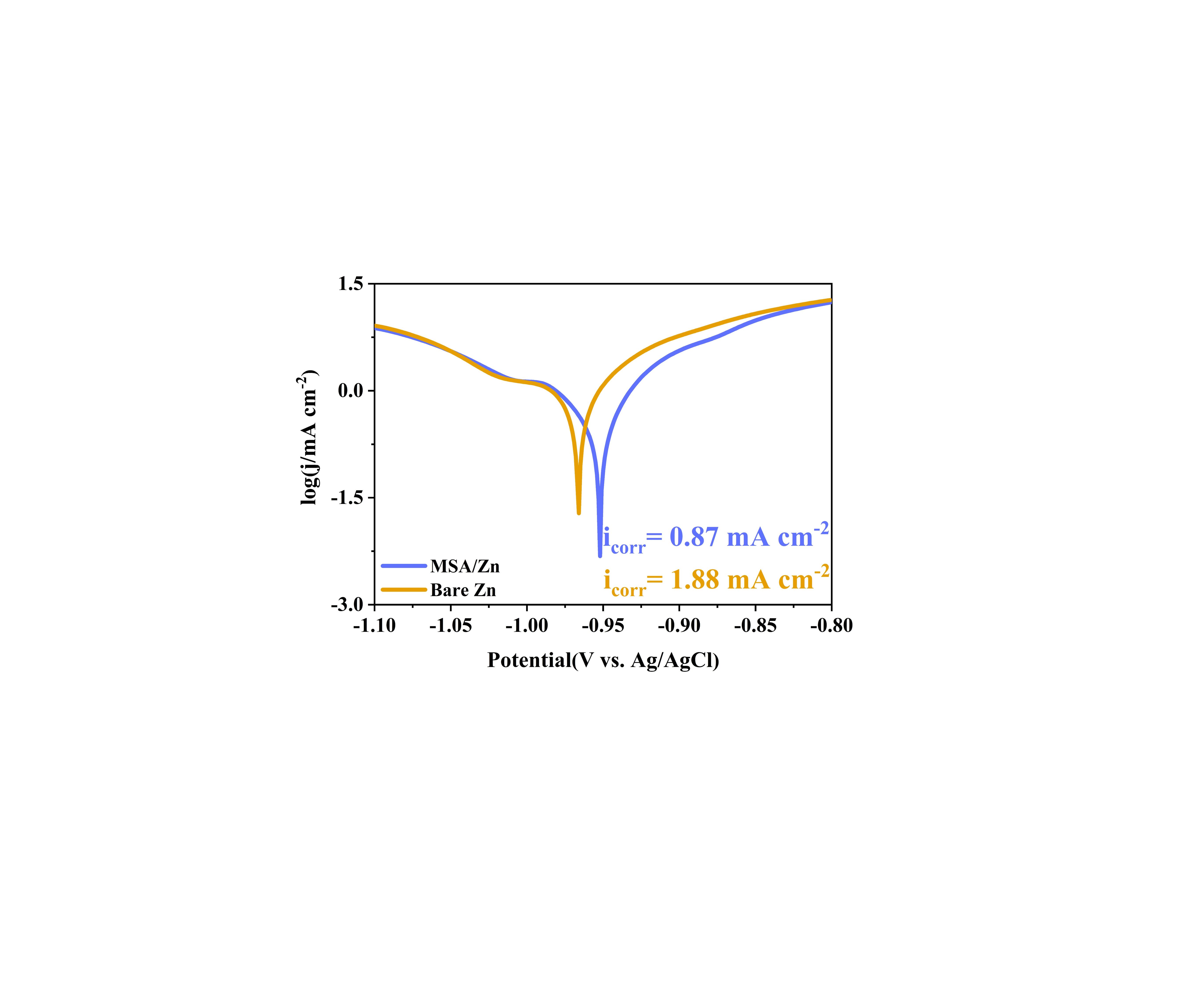
**

**Figure S6.** Tafel plots of Zn||Zn cell with bare Zn and MSA/Zn.

**
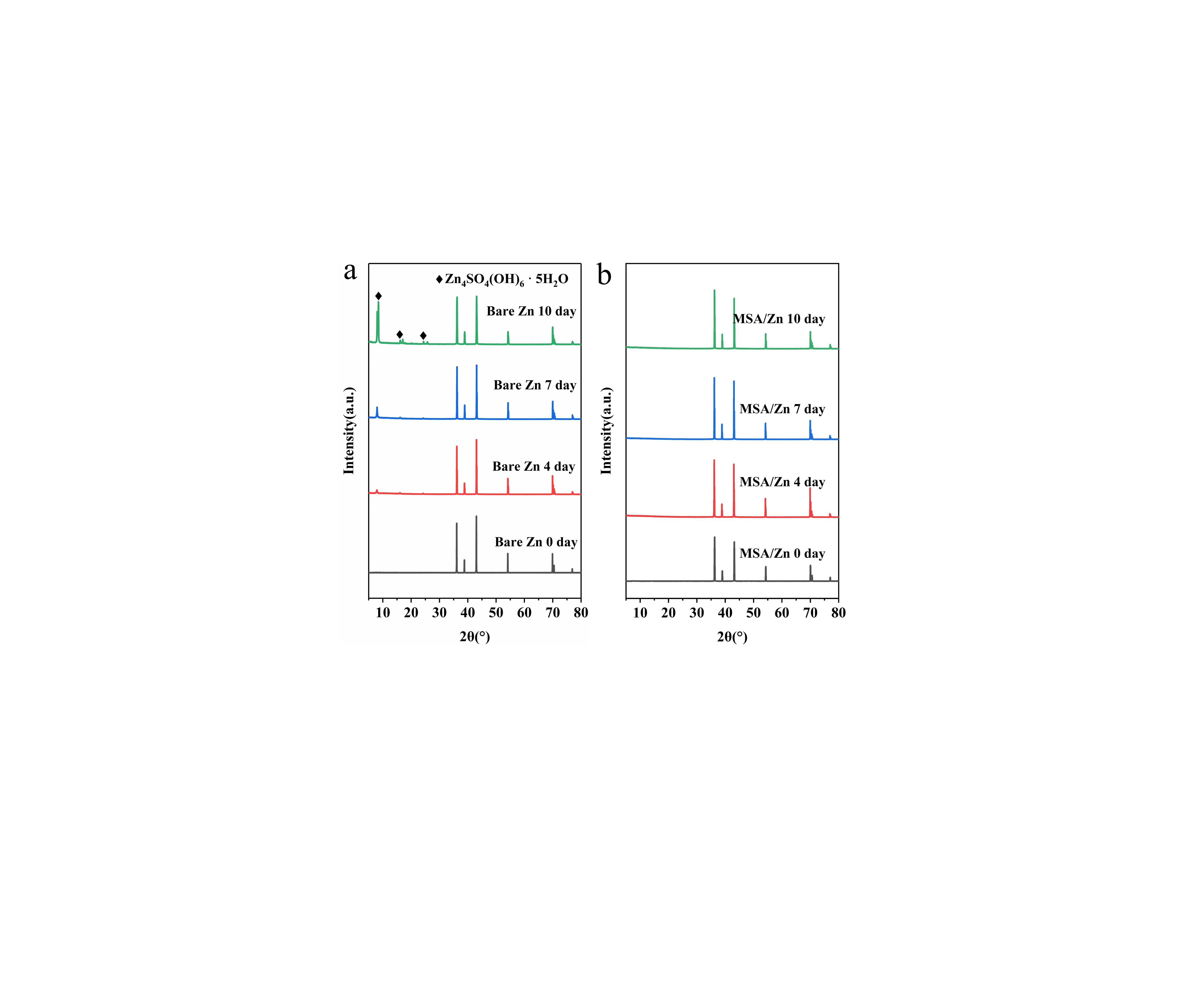
**

**Figure S7.** XRD patterns of bare Zn (c) and MSA/Zn (d) anode in ZnSO_4_ electrolyte.

**
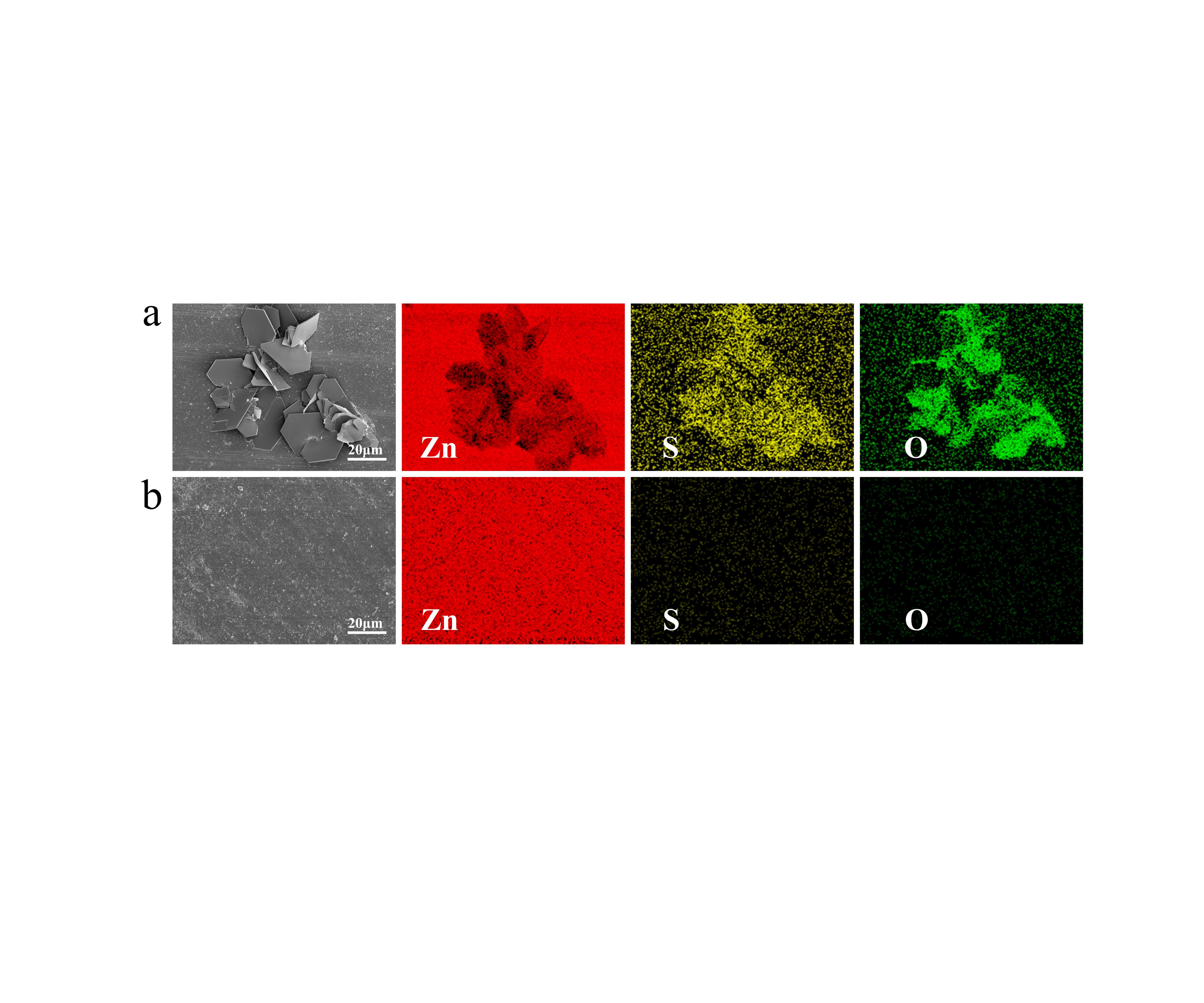
**

**Figure S8.** SEM of bare Zn (a) and MSA/Zn (b) after immersing in ZnSO_4_ electrolyte for 10 days.


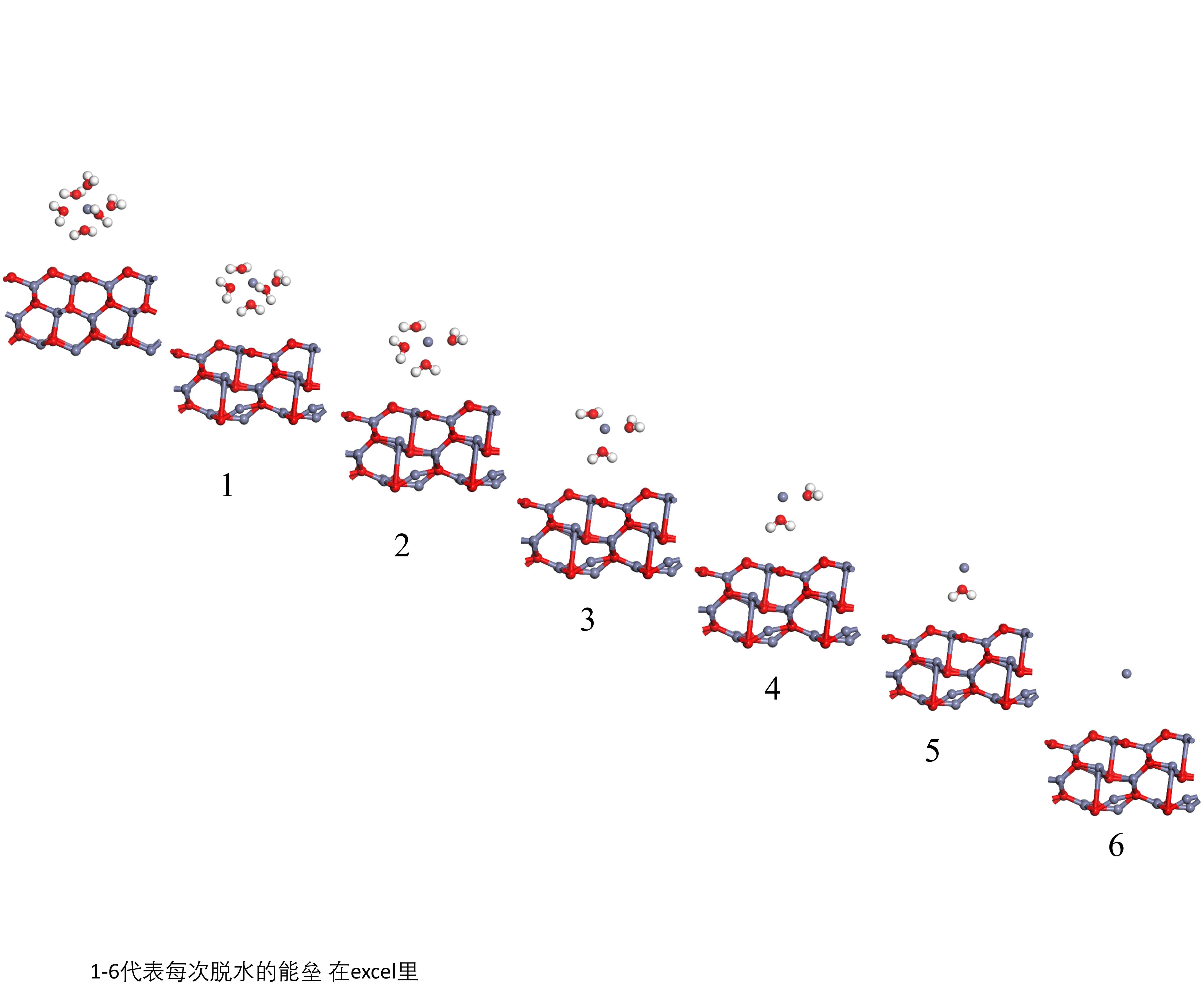


**Figure S9.** Model diagram of the desolvation process of the solvated Zn^2+^ ion in bare Zn anode.


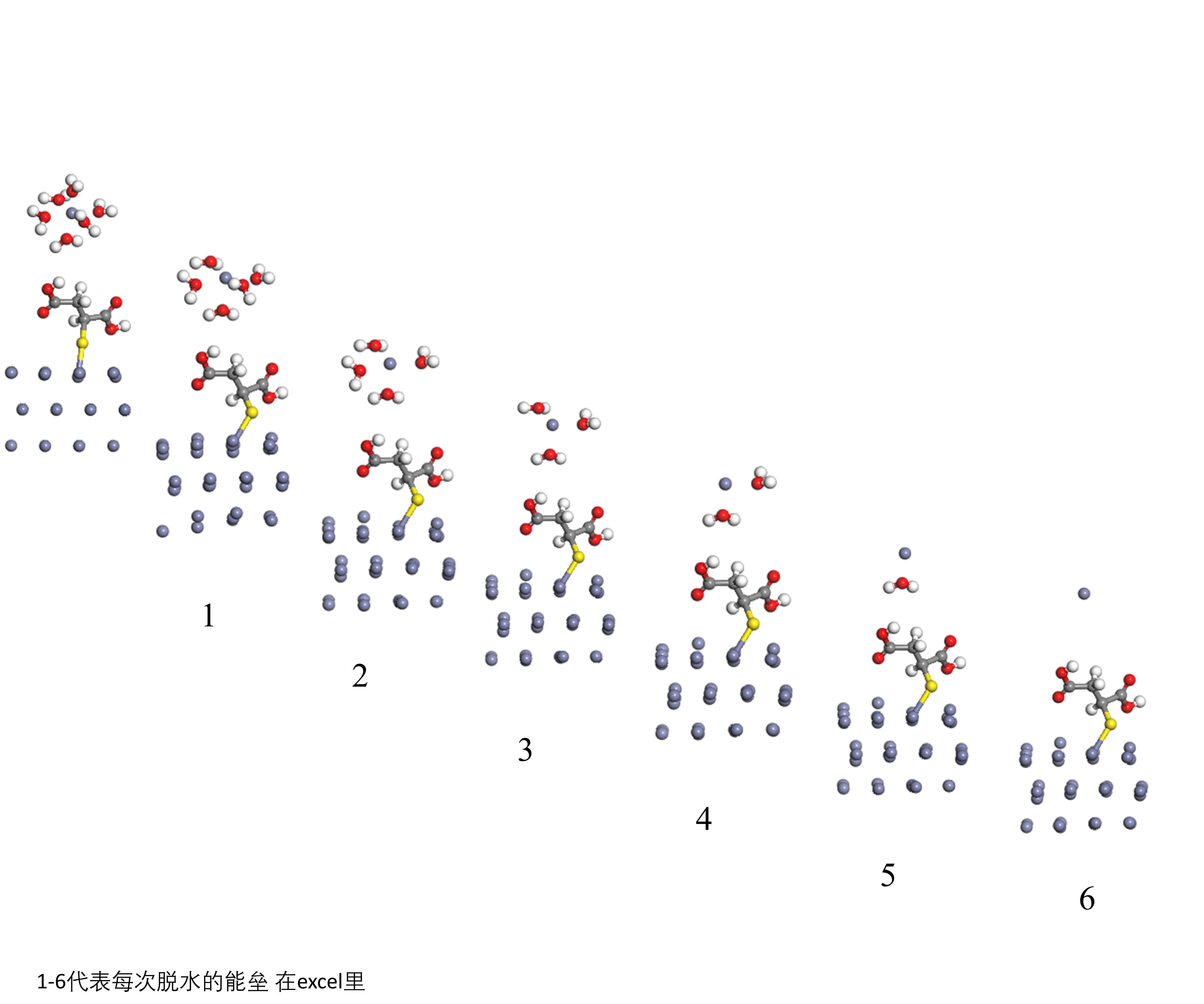


**Figure S10.** Model diagram of the desolvation process of the solvated Zn^2+^ ion in MSA/Zn anode.


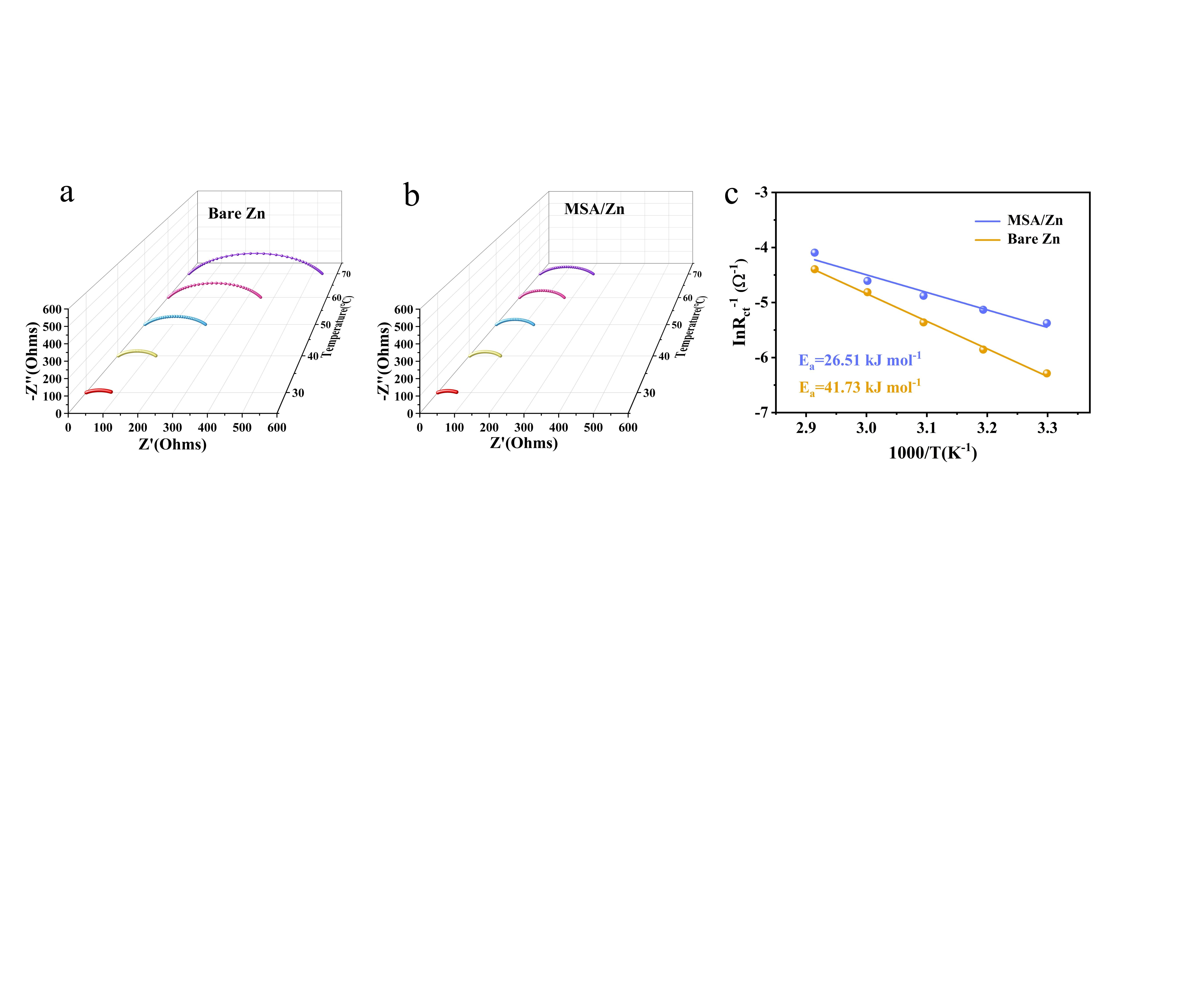


**Figure S11.** Nyquist plots of Zn||Zn and MSA/Zn||MSA/Zn symmetric cell tests at the temperature range of 30 to 70℃. (c) Corresponding ln(*R_ct_*^-1^) versus 1000/T plots revealing desolvation activation energy for Zn^2+^ ion.

**
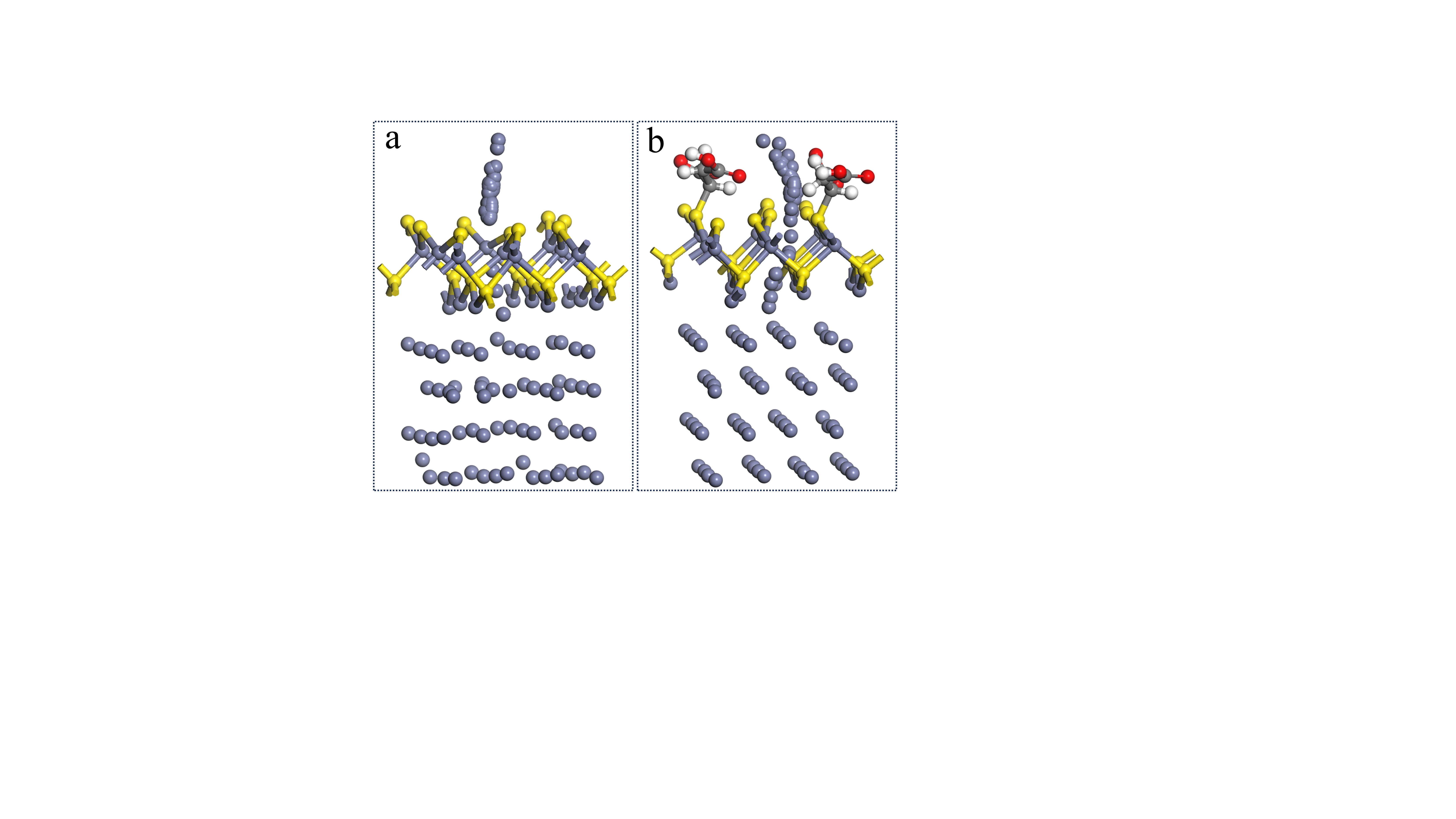
**

**Figure S12.** Model diagram of Zn^2+^ migration barrier in the (a) ZnS/Zn and (b) MSA/Zn electrodes.

**
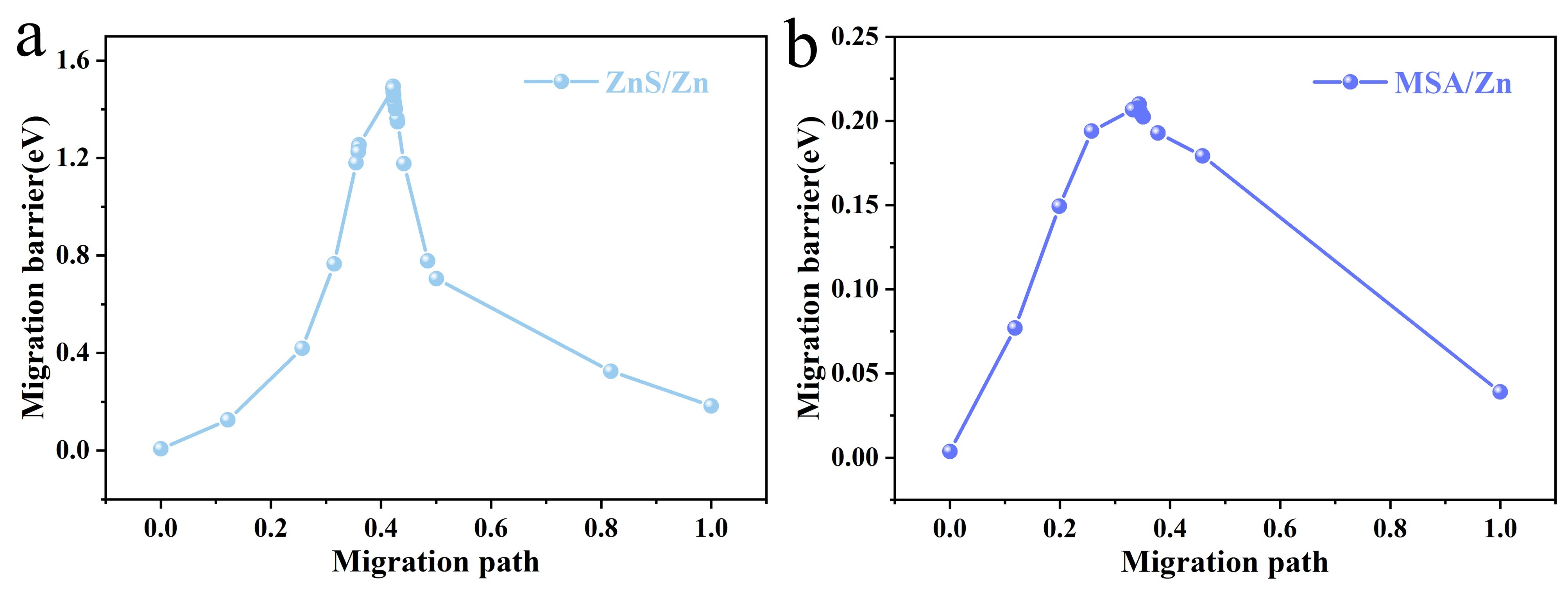
**

**Figure S13.** Zn^2+^ migration barrier in the (a) ZnS/Zn and (b) MSA/Zn electrodes.


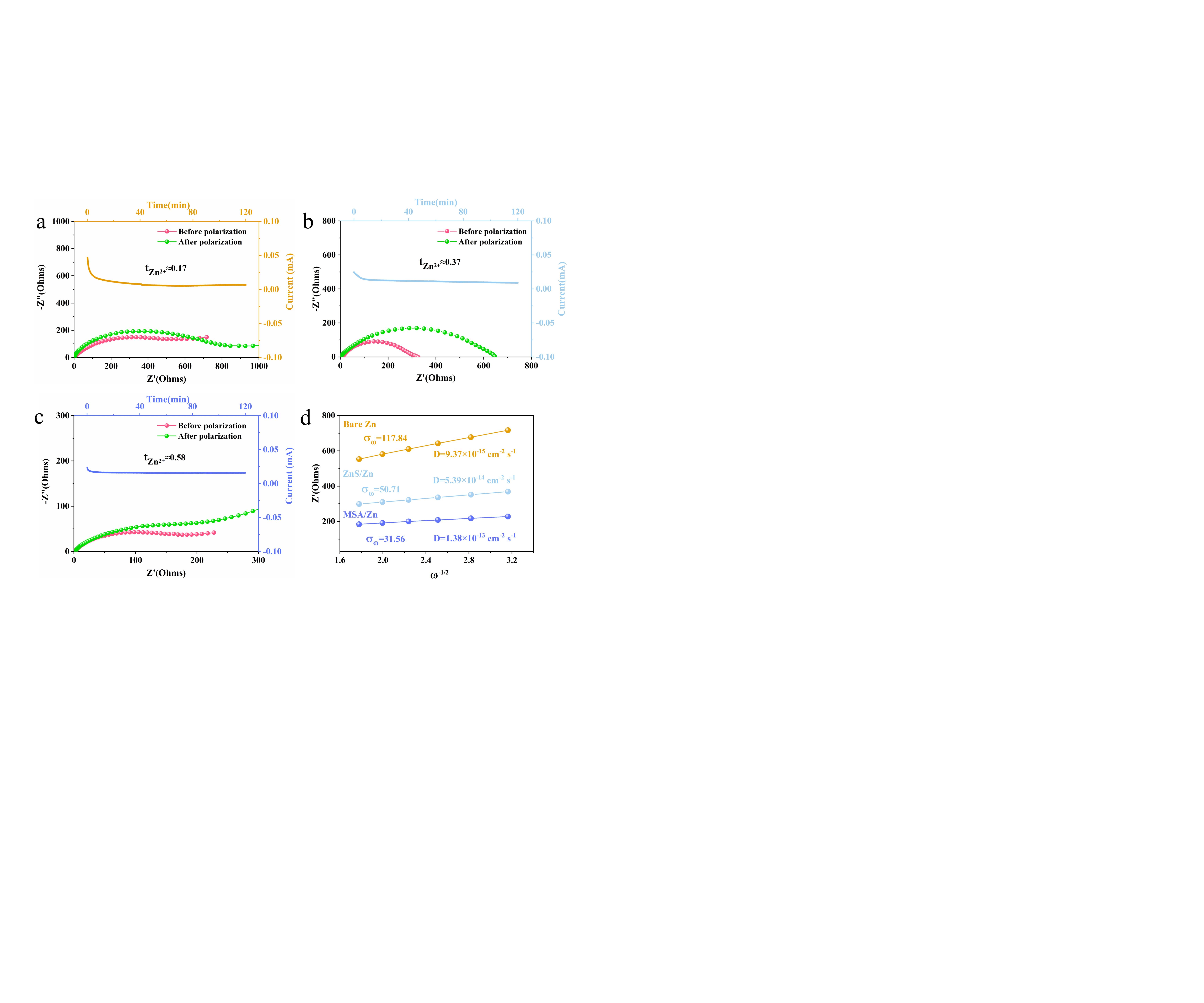


**Figure S14.** *I-t* curve at the bias potential of 10 mV and Nyquist plots before and after polarization of (a) Zn||Zn, (b) ZnS/Zn|| ZnS/Zn, and (c) MSA/Zn||MSA/Zn symmetric cell. (d) Corresponding fitting plots of Z’ vs ω^-1/2^ at lower angular frequencies.


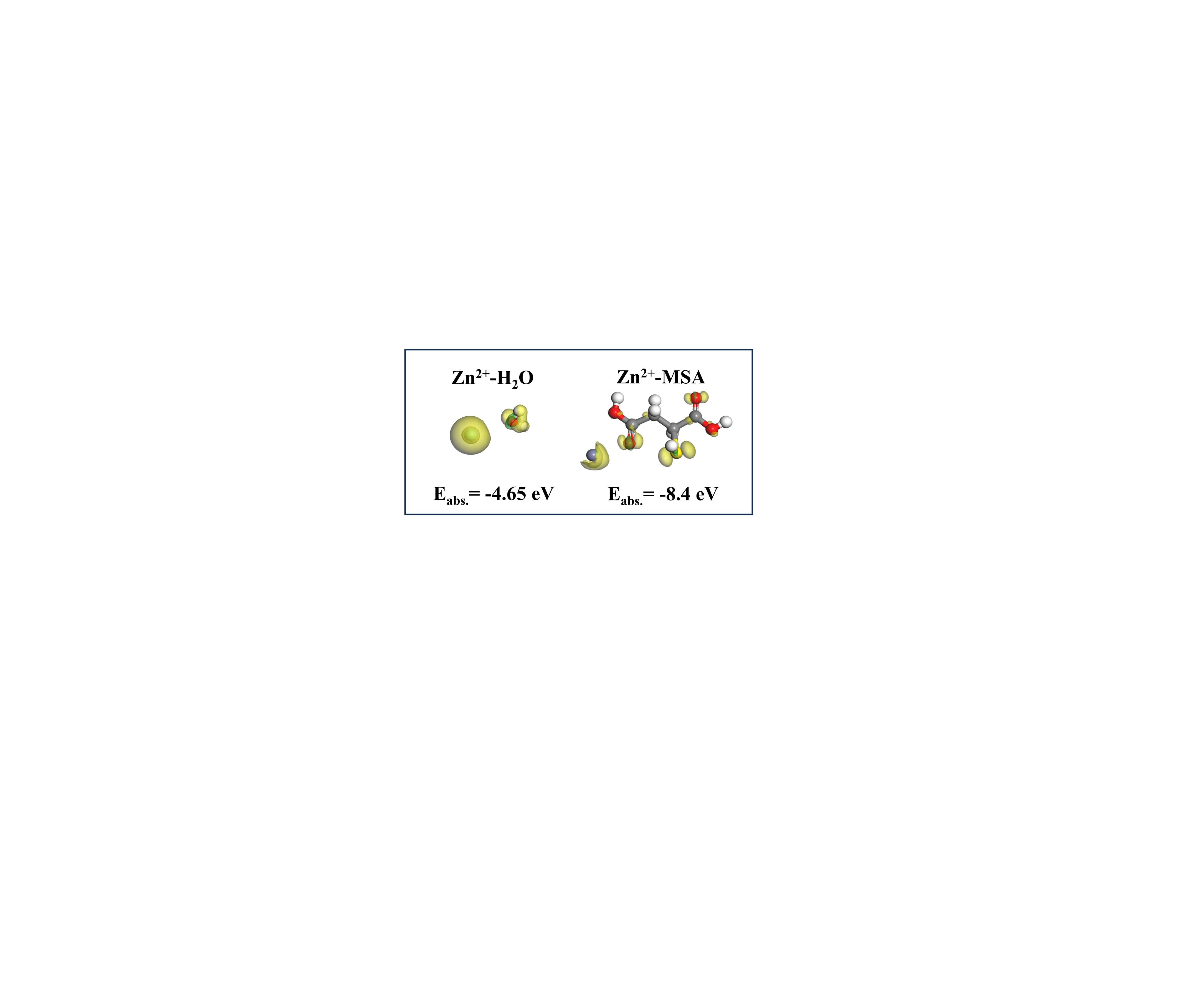


**Figure S15.** The adsorption energy barriers of Zn^2+^ with H_2_O and MSA.


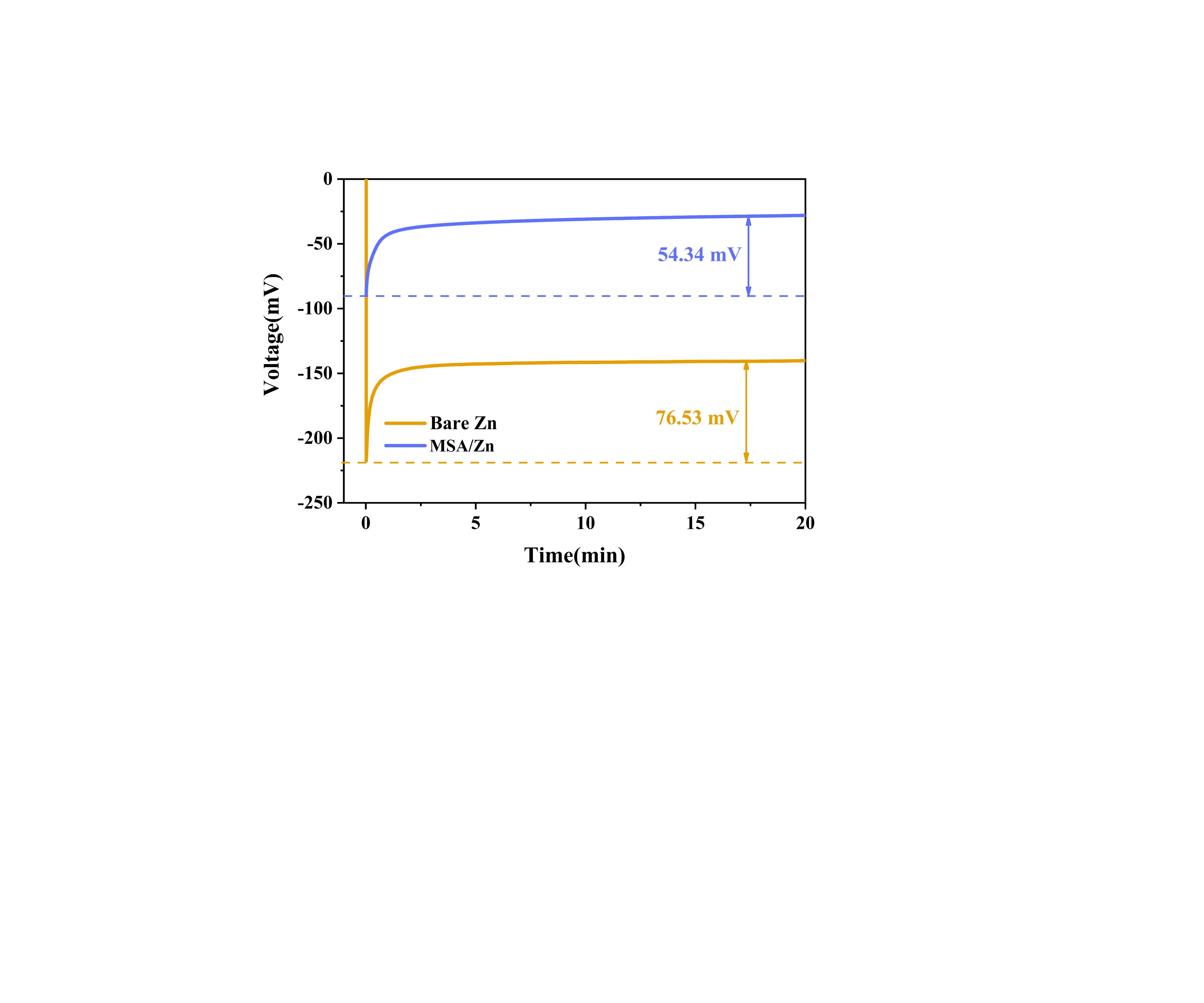


**Figure S16.** Nucleation overpotential of Zn||Zn and MSA/Zn||MSA/Zn


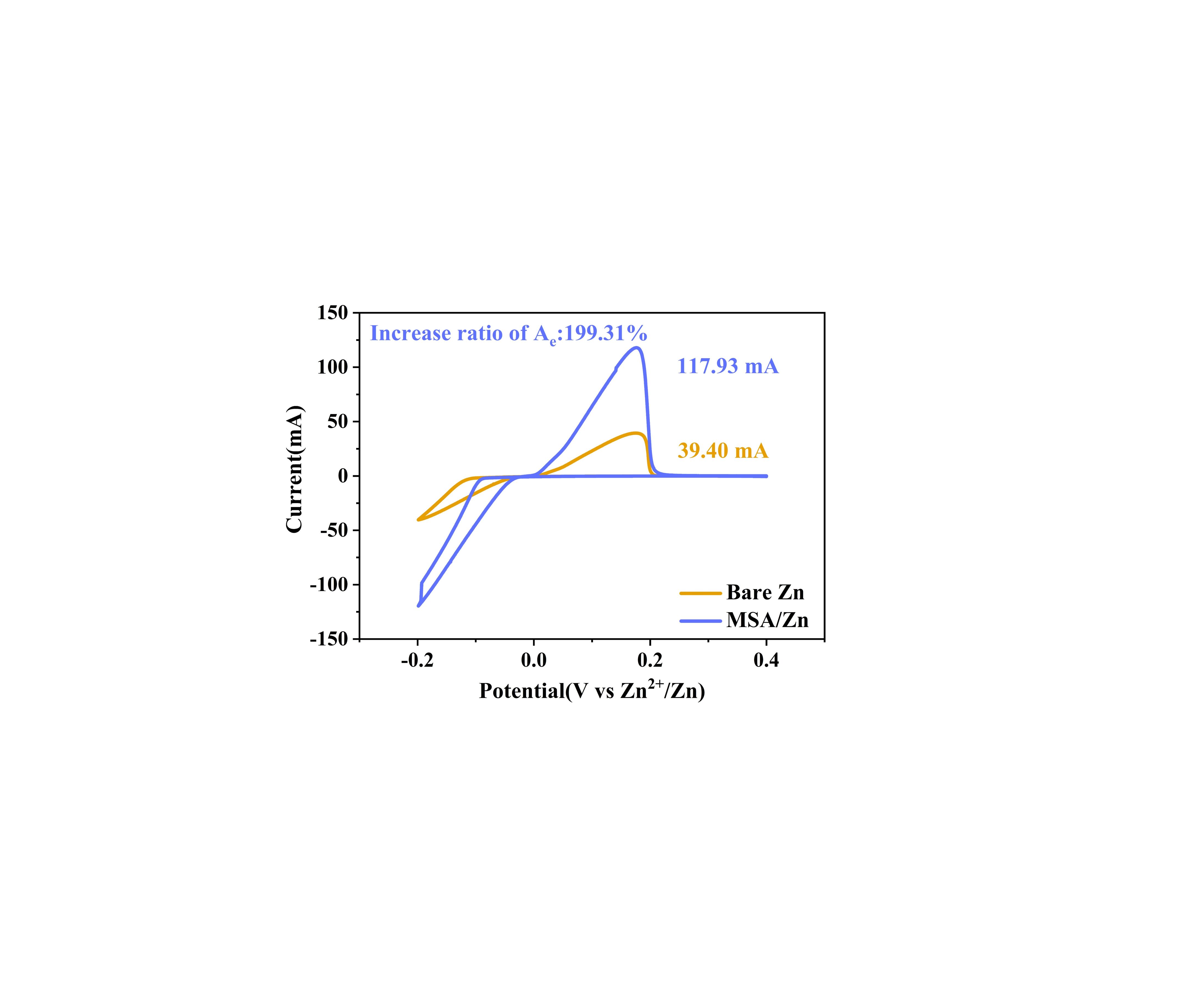


**Figure S17.** CV curves of Zn||SS and MSA/Zn||SS.

**
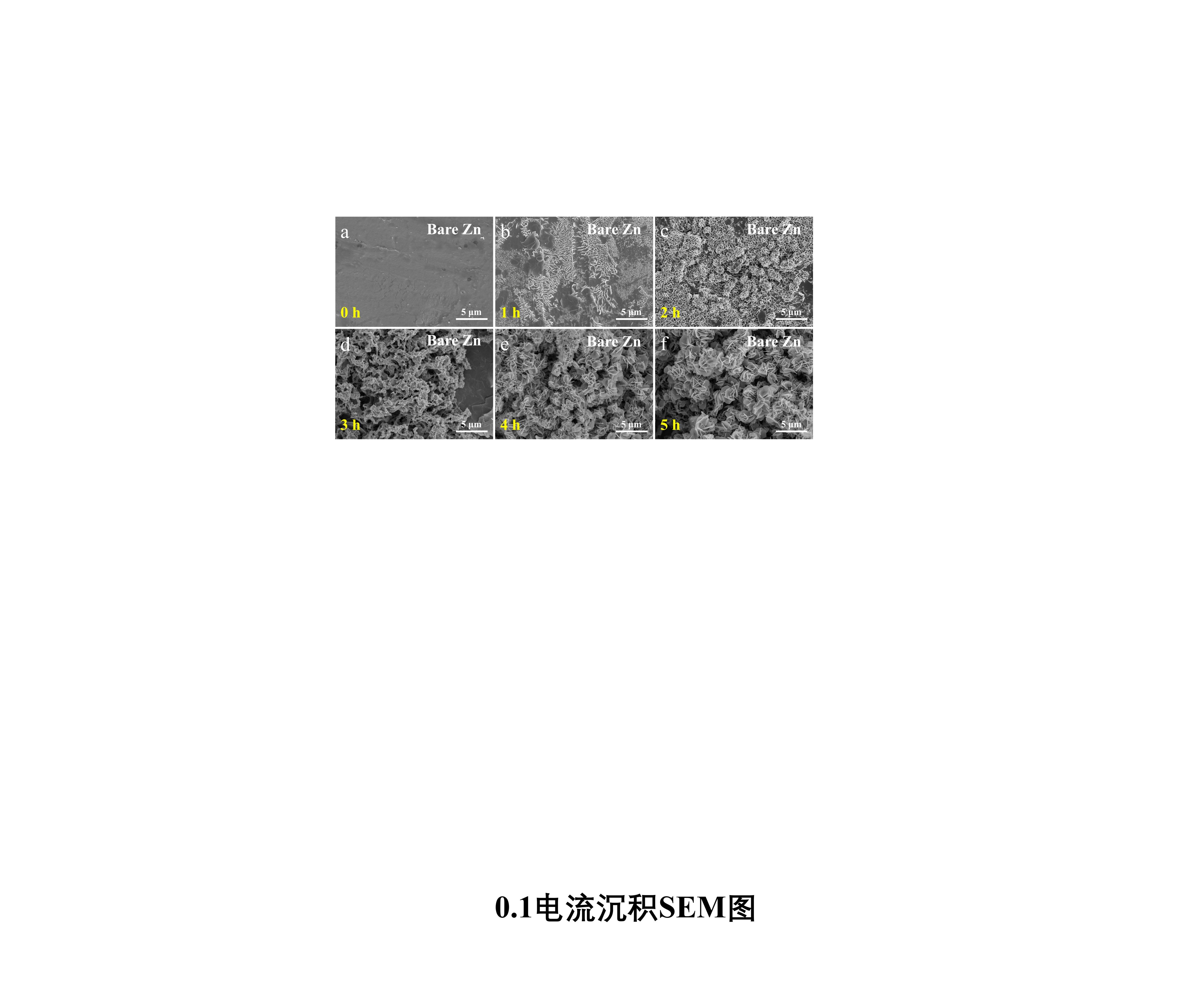
**

**Figure S18.** SEM images of bare Zn after 0, 1, 2, 3, 4 and 5 hours of deposition at the current density of 0.1 mA cm^-2^.

**
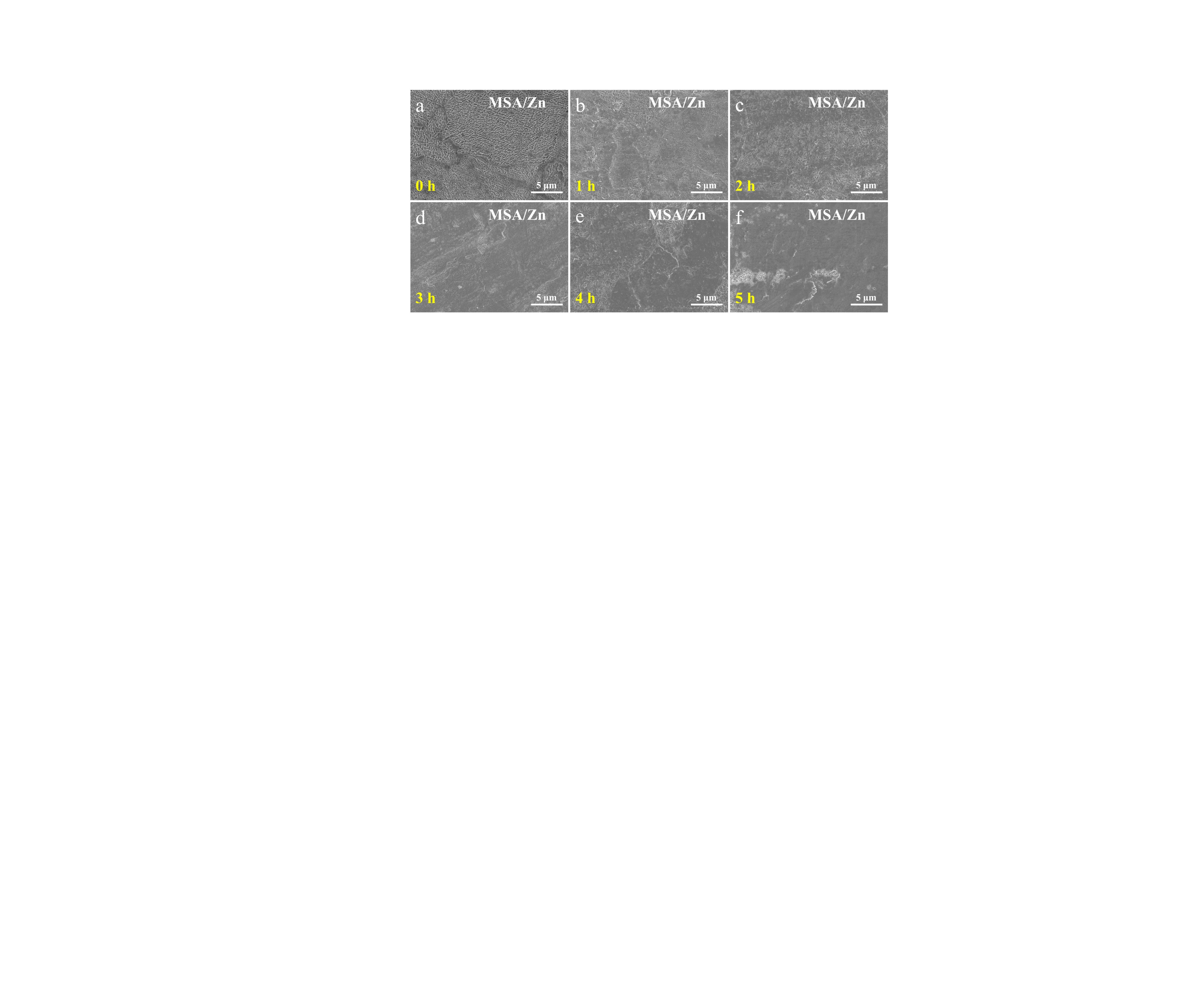
**

**Figure S19.** SEM images of MSA/Zn after 0, 1, 2, 3, 4 and 5 hours of deposition at the current density of 0.1 mA cm^-2^.

**
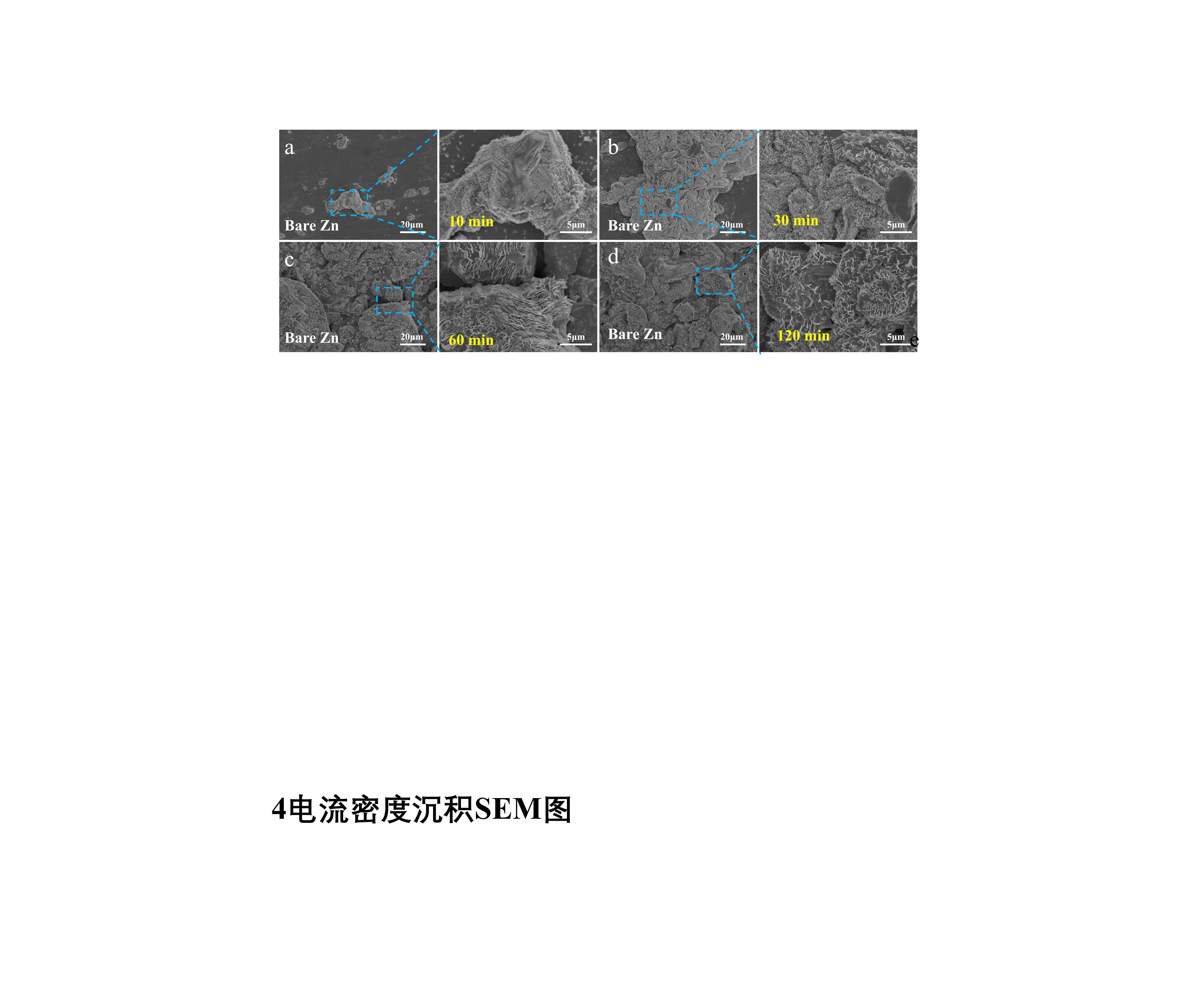
**

**Figure S20.** SEM images of bare Zn after 10, 30, 60, and 120 minutes of deposition at the current density of 4 mA cm^-2^.

**
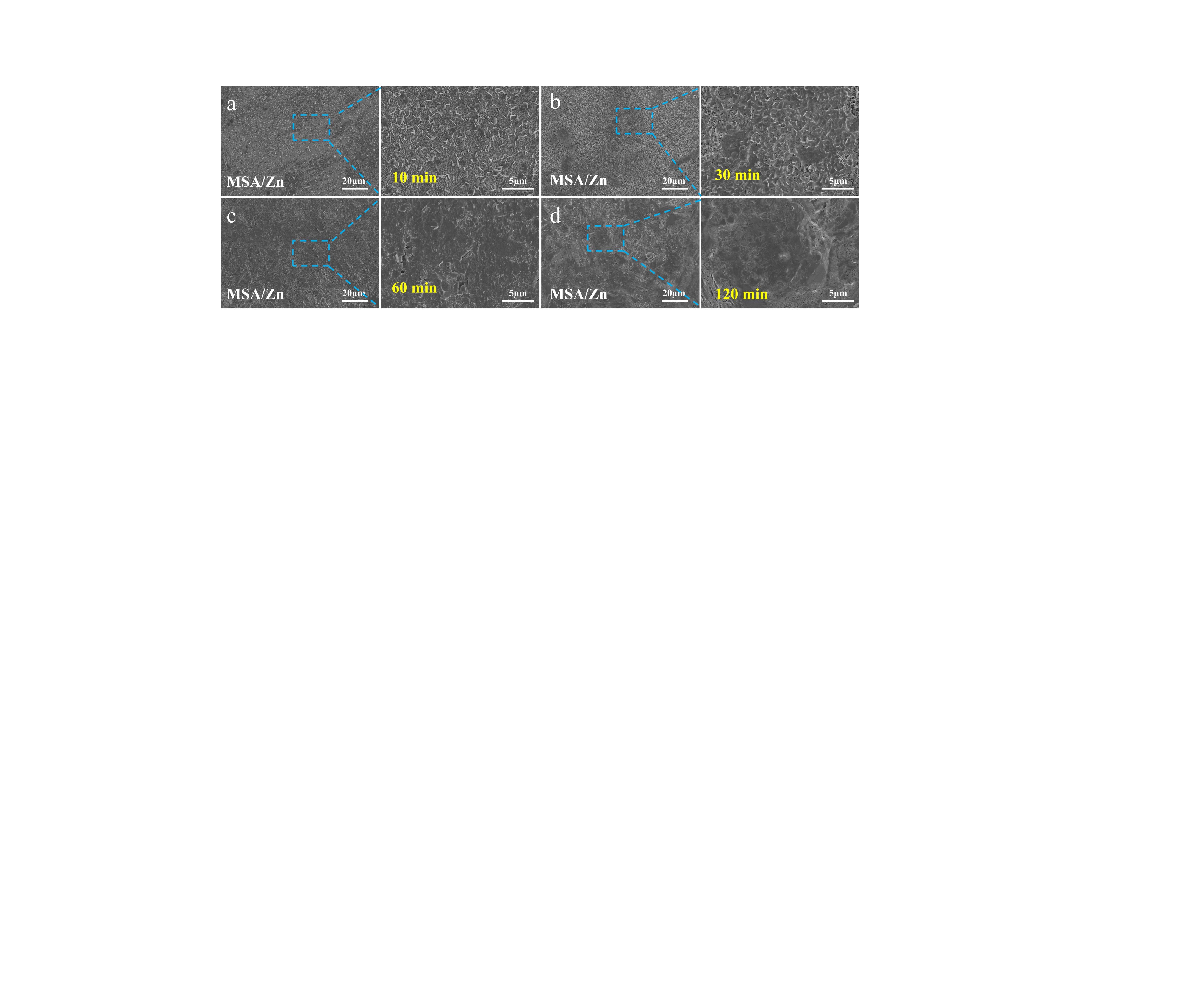
**

**Figure S21.** SEM images of MSA/Zn after 10, 30, 60, and 120 minutes of deposition at the current density of 4 mA cm^-2^.

**
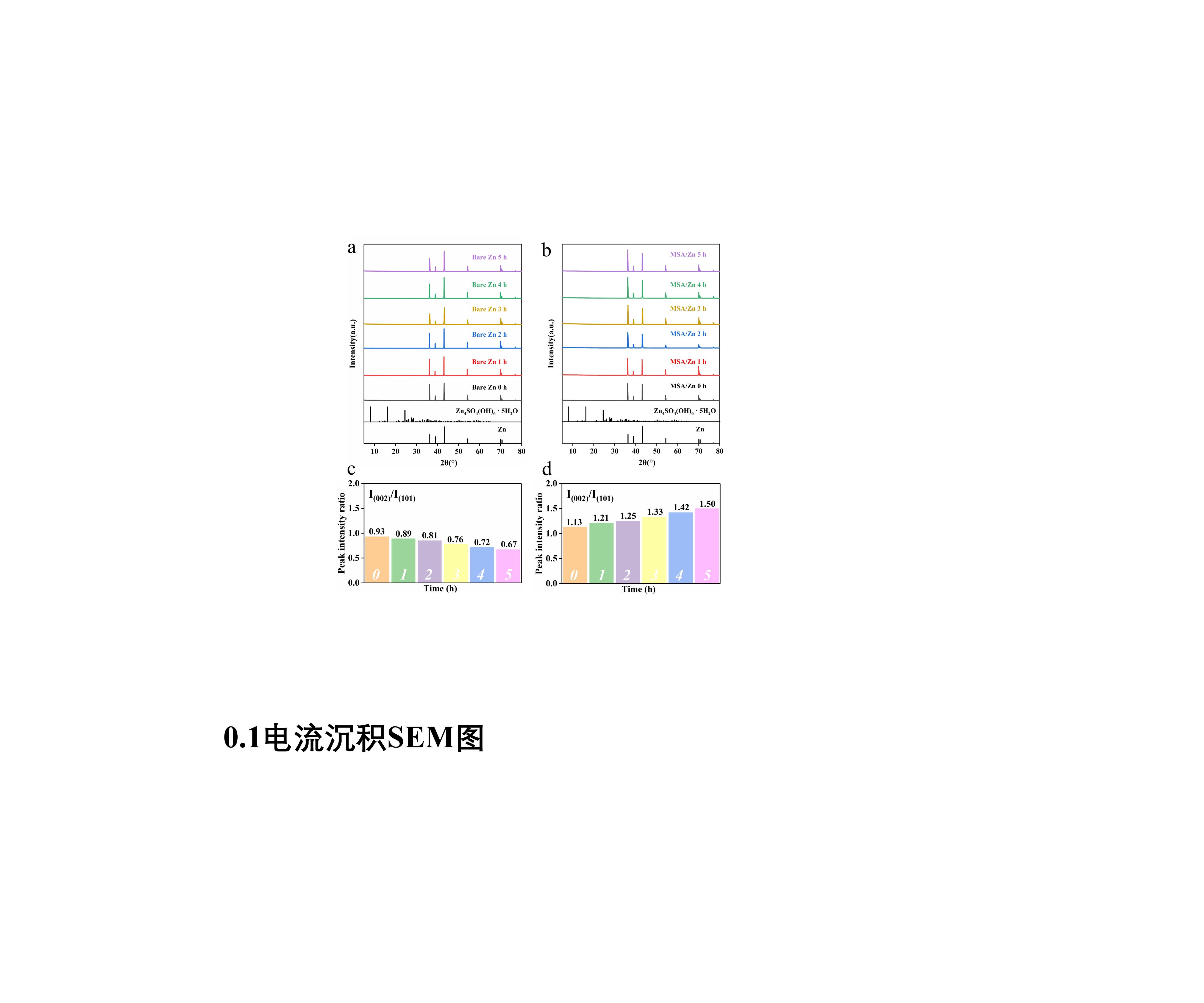
**

**Figure S22.** XRD patterns (a, b) and facet ratio (c, d) of bare Zn (a, c) and MSA/Zn (b, d) after deposition at the current density of 0.1 mA cm^-2^.

**
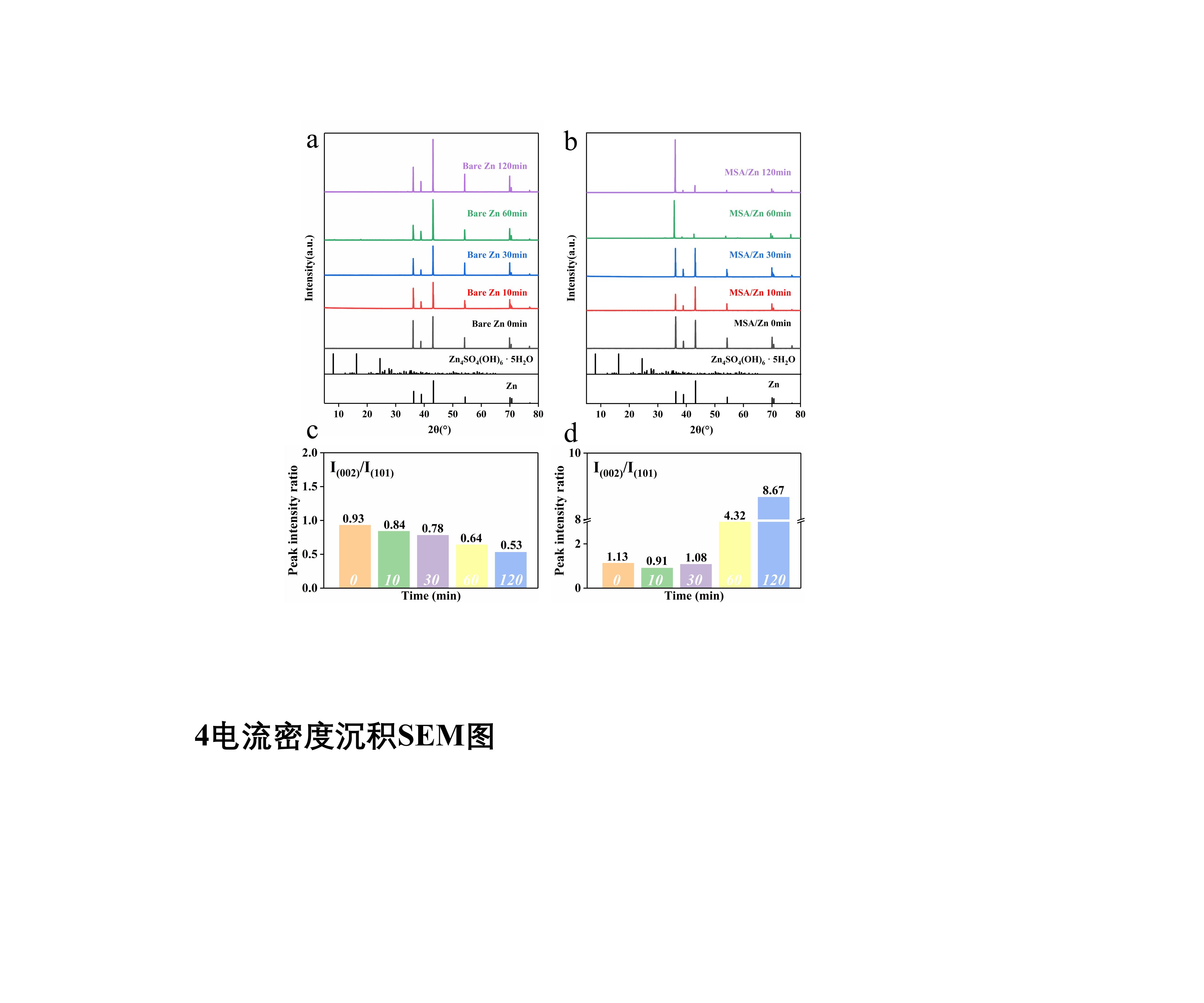
**

**Figure S23.** XRD patterns (a, b) and facet ratio (c, d) of bare Zn (a, c) and MSA/Zn (b, d) after deposition at the current density of 4 mA cm^-2^.

**
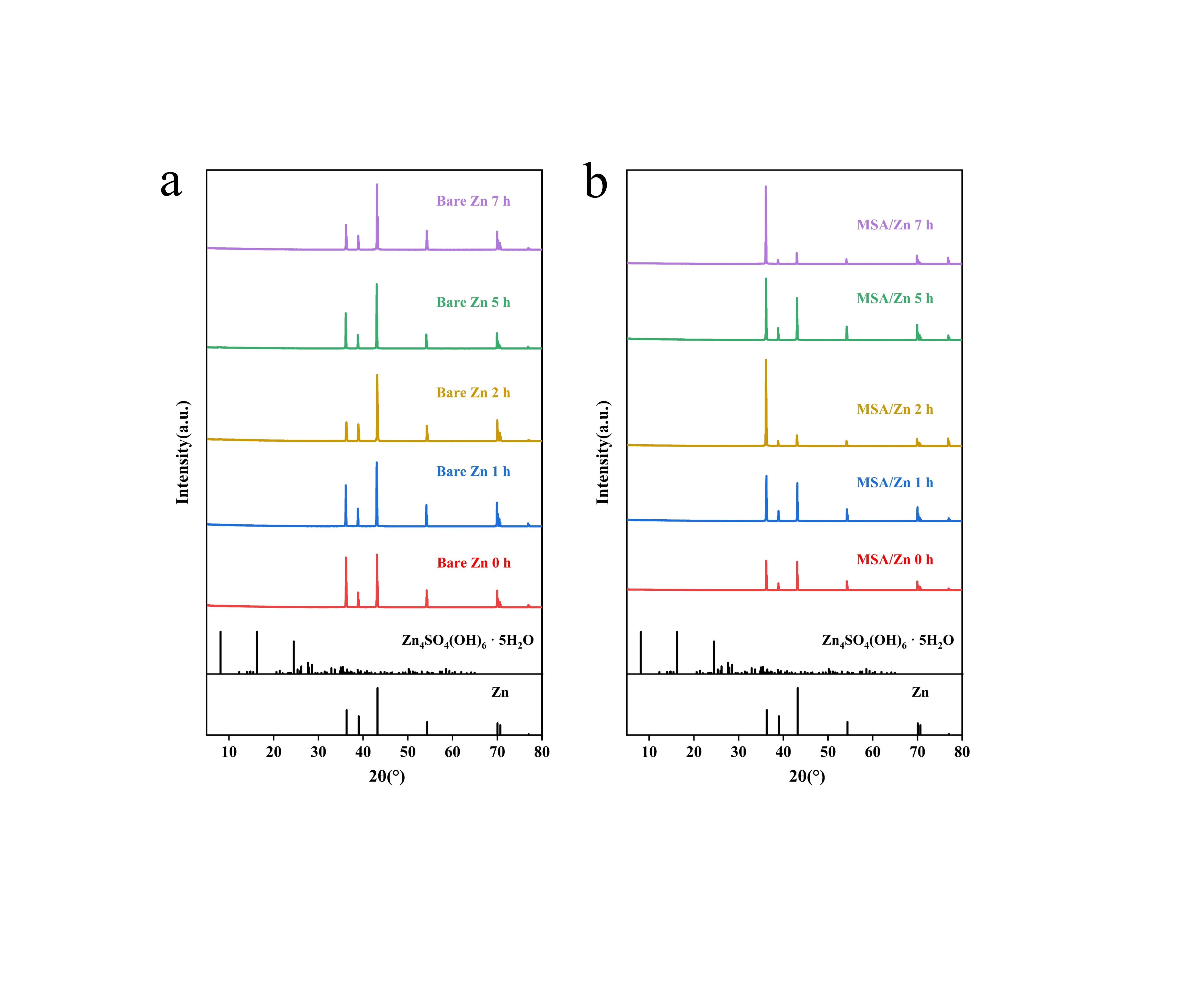
**

**Figure S24.** The XRD patterns of bare Zn (a) and MSA/Zn (b) during the plating process at the current density of 1 mA cm^-2^.

**
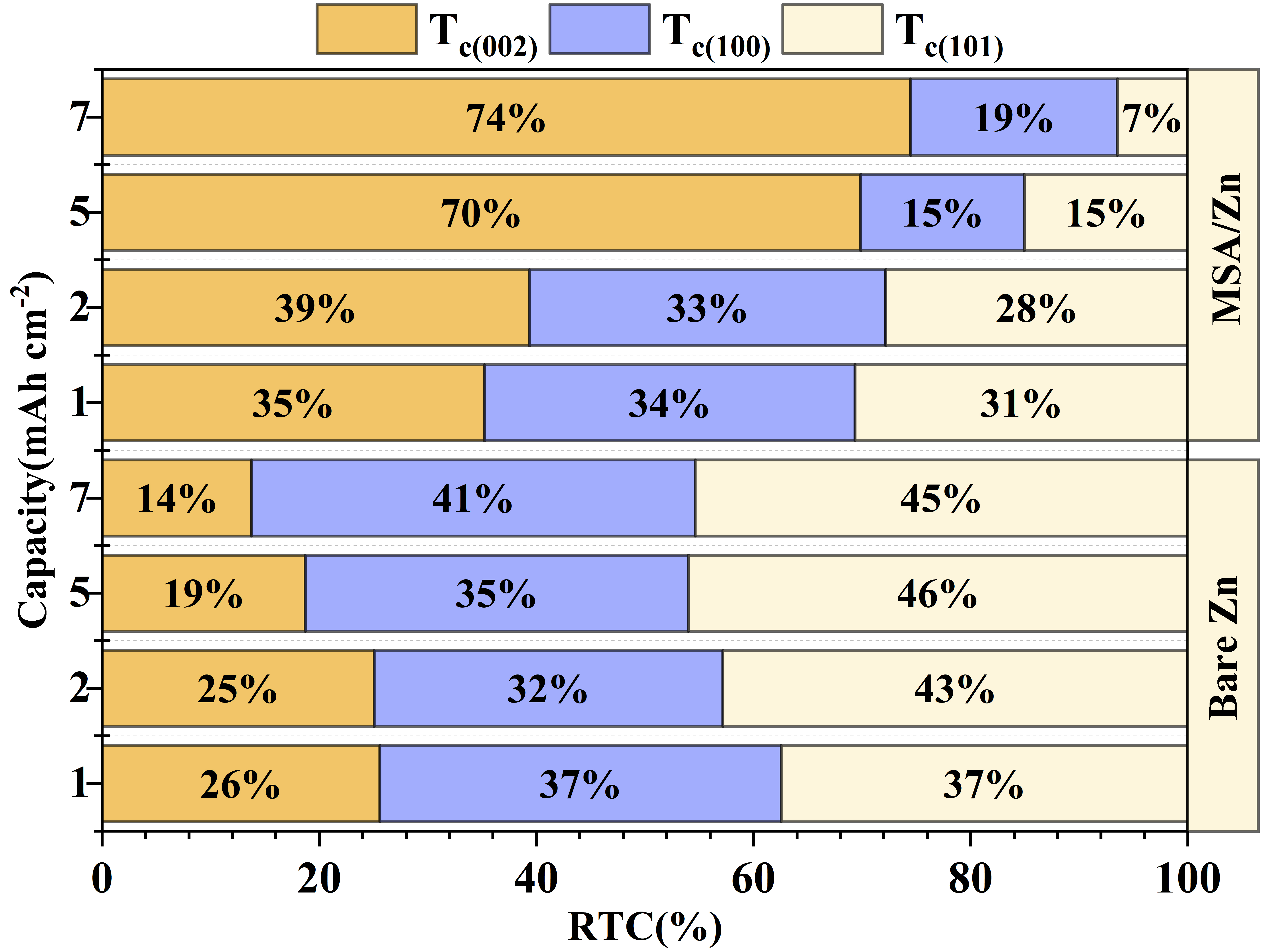
**

**Figure S25.** RTC of bare Zn and MSA/Zn corresponding XRD patterns.

**
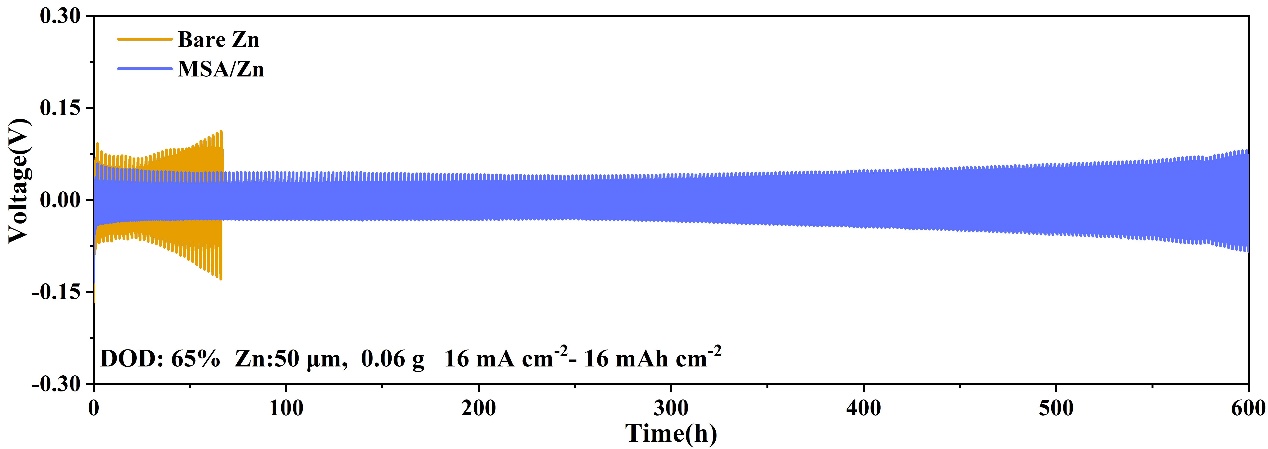
**

**Figure S26.** DOD profiles in bare Zn and MSA/Zn anodes: 16 mA cm^-2^ and capacity of 16 mAh cm^-2^.

**
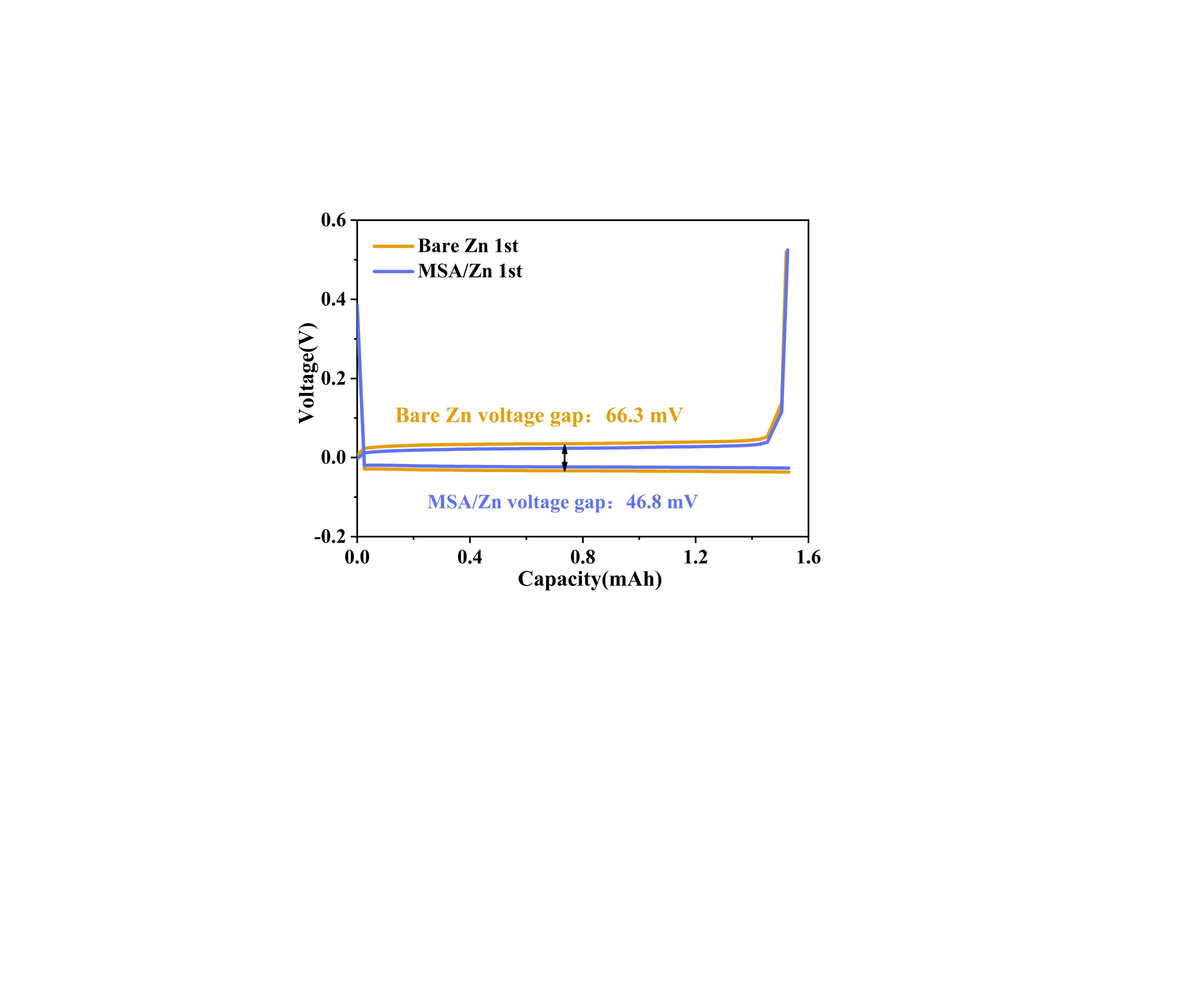
**

**Figure S27.** The voltage profiles of Zn||Cu and MSA/Zn||Cu.

**
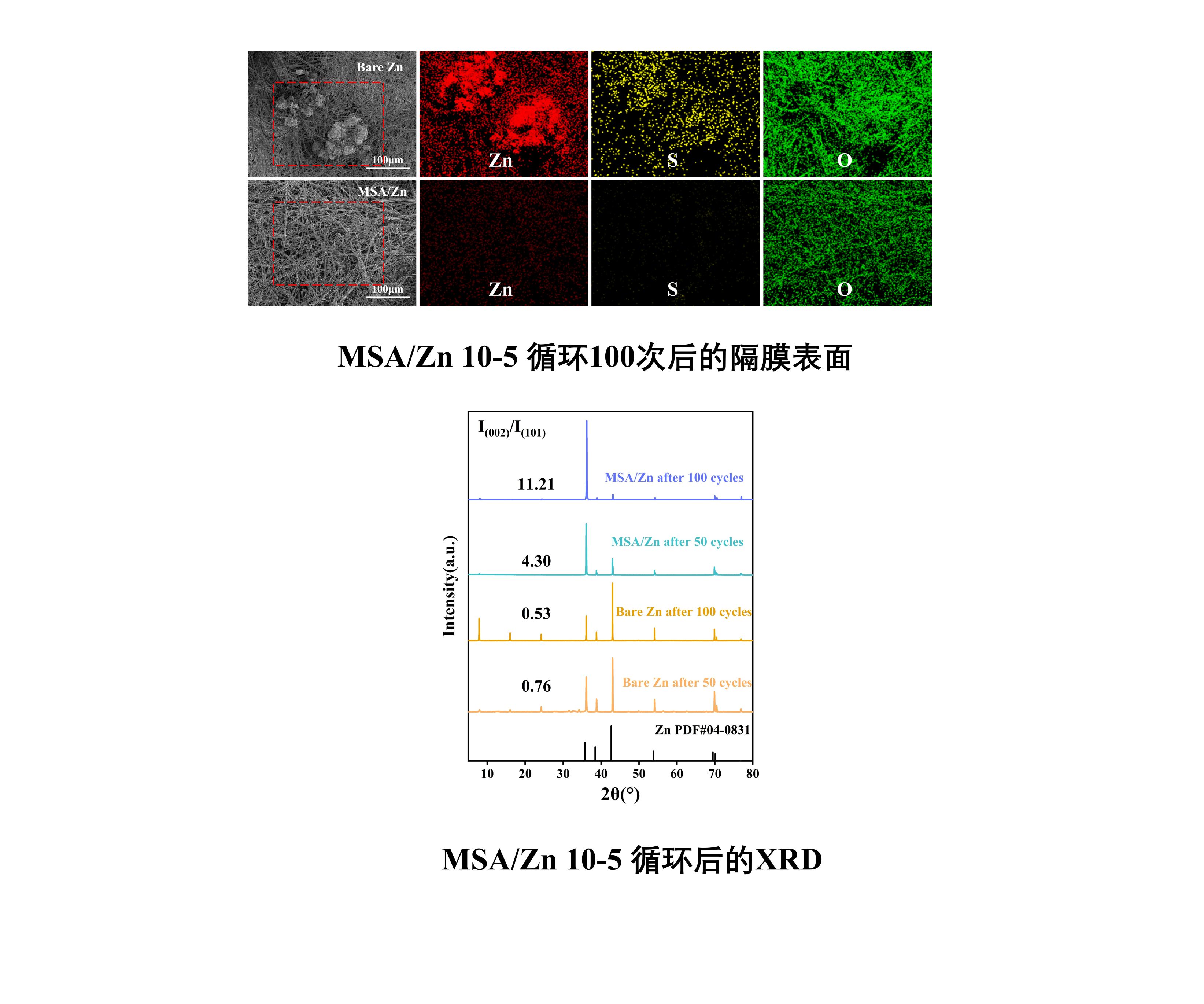
**

**Figure S28.** XRD patterns of bare Zn and MSA/Zn after 50 and 100 cycles at 10 mA cm^-2^, 5 mAh cm^-2^.

**
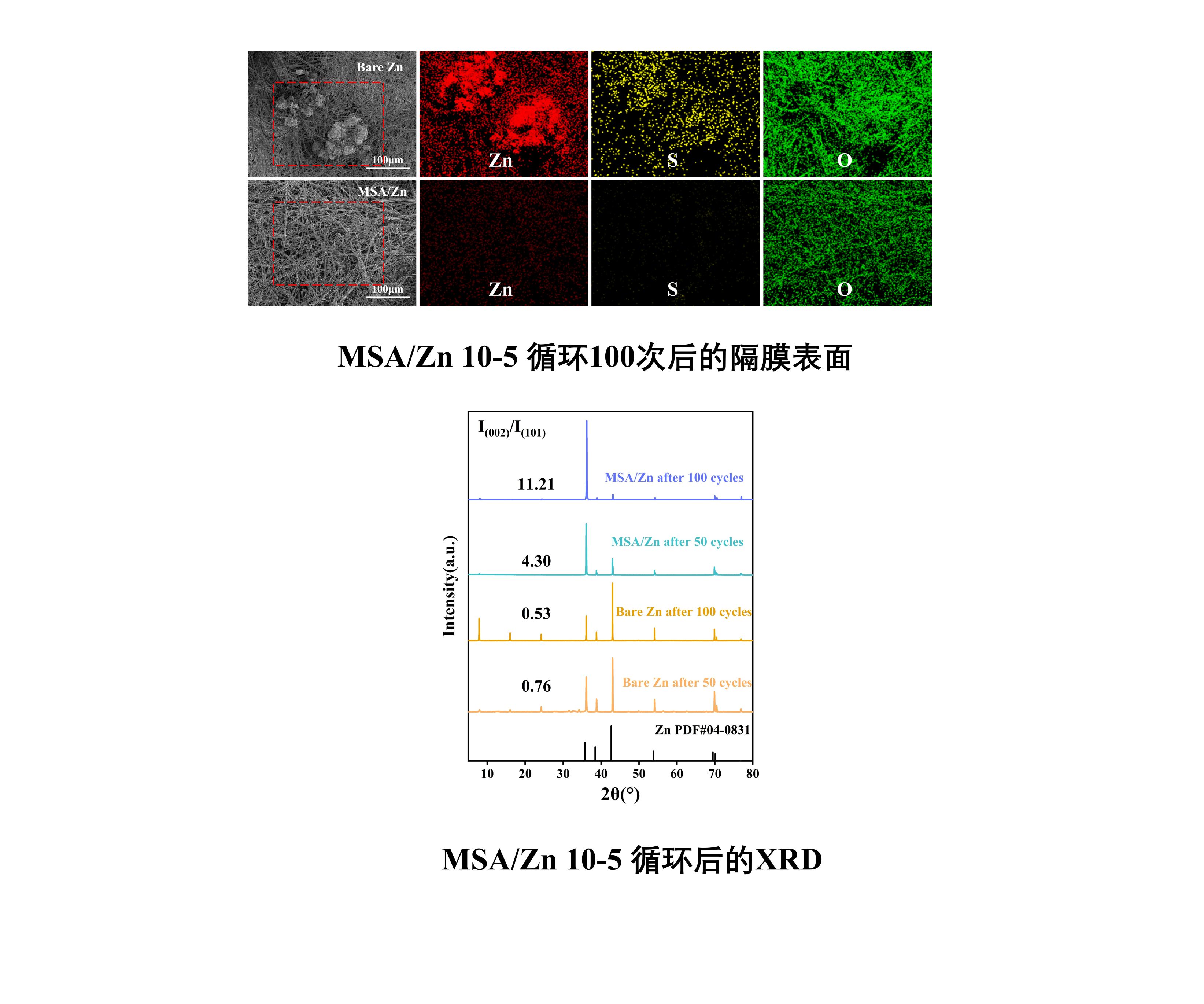
**

**Figure S29.** Surface and corresponding Zn, O and S elemental maps of the membrane of the symmetrical cell with bare Zn and MSA/Zn at 10 mA cm^-2^, 5 mAh cm^-2^.

**
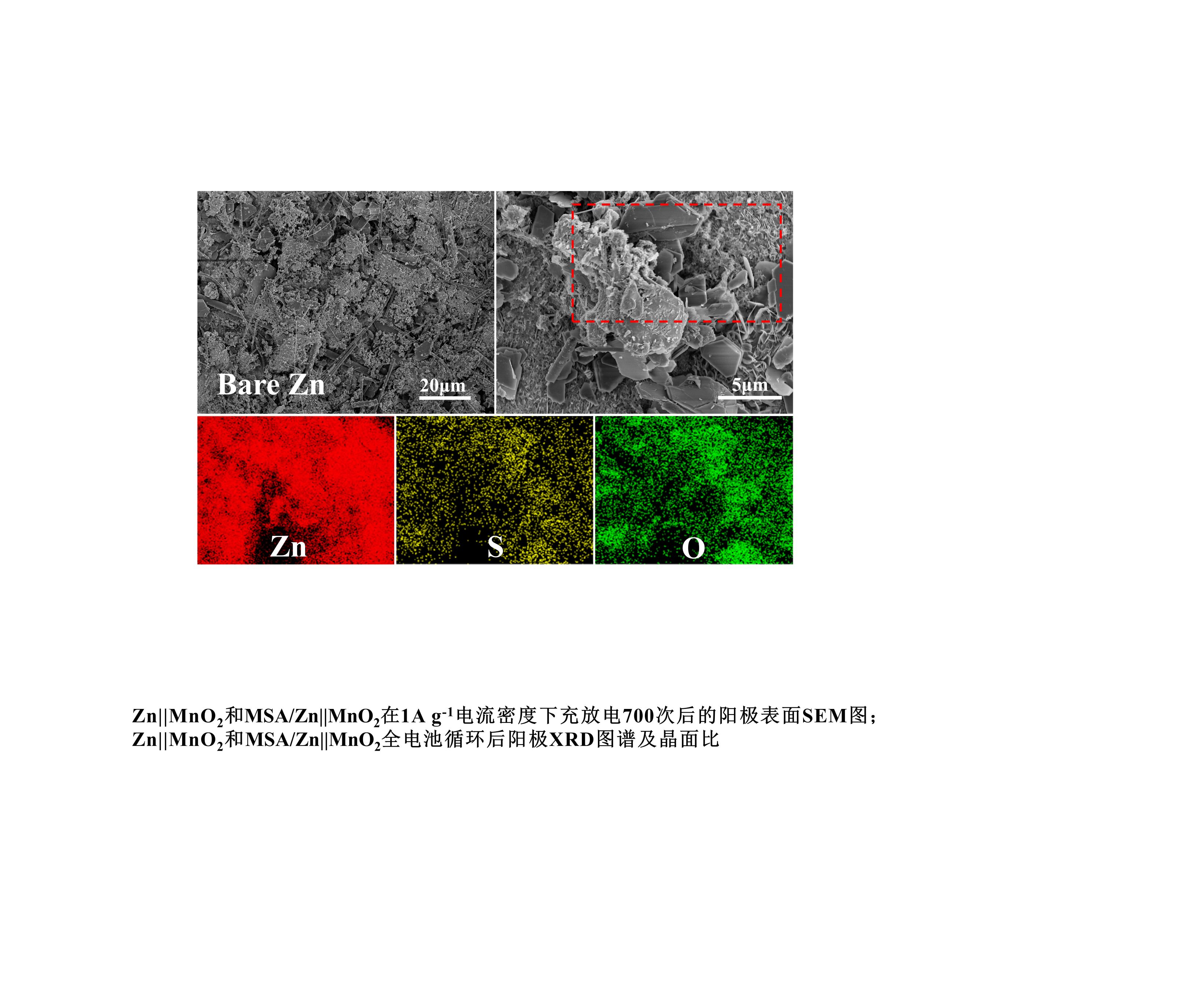
**

**Figure S30.** SEM images of bare Zn after 400 cycles and elemental mappings.

**
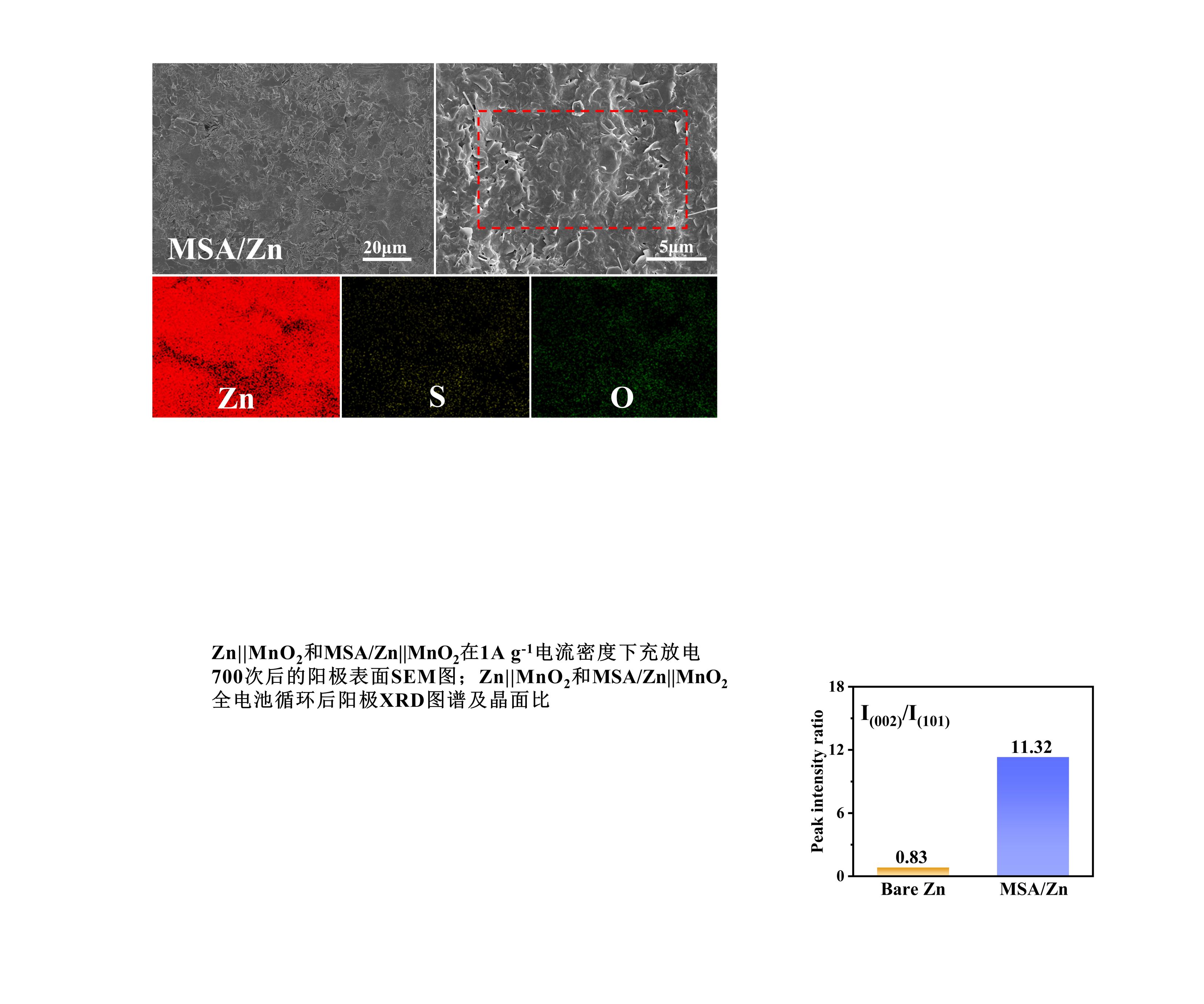
**

**Figure S31.** SEM images of MSA/Zn after 400 cycles and elemental mappings.

**Table S1.** Comparison of cycling performance for this work with recently reported Zn||Zn symmetric cells in Figure 4e.

| Anode | Current density  (mA cm^-2^) | Areal capacity  (mAh cm^-2^) | Cyclic life(h) | Reference |
| --- | --- | --- | --- | --- |
| ZHS-Zn(200) | 4 | 2 | 1000 | [38] |
|  | 10 | 5 | 400 |  |
| 3DH-Zn | 5 | 1 | 2000 | [39] |
| ZIF@Crwon@Zn | 2  1 | 1  0.5 | 1900  1300 | [40] |
| Ti_4_O_7_@Zn | 10 | 1 | 2100 | [41] |
| ZnSO_4_ + K-PAM electrolyte | 1 | 1 | 1200 | [42] |
| C@RZn | 1 | 1 | 2000 | [43] |
|  | 5 | 1 | 1800 |  |
| DPM-Zn | 1 | 0.5 | 1600 | [44] |
| ZCL2 | 2 | 0.5 | 500 | [45] |
| Org-Cu@Zn | 5 | 5 | 1350 | [46] |
| ZnSA@Zn | 5 | 1 | 800 | [47] |
| NTP@TPEU/Zn | 2 | 1 | 1670 | [48] |
|  | 10 | 1 | 500 |  |
| ZnSO_4_+melamine electrolyte | 1 | 1 | 1100 | [49] |
| 2D@Zn | 1  5 | 1  5 | 1000  700 | [50] |
| Zn@CaSi | 1 | 0.25 | 1580 | [51] |
| ZnSnO_3_@Zn | 2  10 | 1  5 | 1124  120 | [52] |
| Zn@ZCO | 4 | 1 | 2100 | [53] |
| MSA/Zn | 10 | 2.5 | 800 | This work |
|  | 22.66 | 11.33 | 180 |  |
|  | 10 | 5 | 2400 |  |
|  | 16 | 16 | 600 |  |
|  | 20 | 20 | 528 |  |

**References**

1. Z. Cheng, K. Wang, J. Fu, F. Mo, P. Lu, J. Gao, D. Ho, B. Li, H. Hu, *Adv*. *Energy Mater*. 2024, *14*, 2304003.
2. P. Wang, S. Liang, C. Chen, X. Xie, J. Chen, Z. Liu, Y. Tang, B. Lu, J. Zhou, *Adv*. *Mater*. **2022**, *34*, 2202733.
3. S. Cao, T. Zhou, Y. Liu, W. Lu, A. Zhang, S. He, P. Yi, L. Ma, Z. Liu, F. Zuo, L. Cao, Z. Ren, M. Ye, H. Fang, J. Shen, *J*. *Am*. *Chem*. *Soc*. 2025, *147*, 36626.
4. H. Cheng, C. Chen, J. Zhang, R. Gui, S. Liu, W. Zhang, J. Peng, Y. Zhu, M. Zhou, W. Chu, Y. Xie, C. Wu, *Adv*. *Mater*. 2025, e16149.
5. B. Xie, Q. Hu, X. Liao, X. Zhang, H. Lang, R. Zhao, Q. Zheng, Y. Huo, J. Zhao, D. Lin, X. Wu, *Adv*. *Funct*. *Mater*. **2024**, *34*, 2311961.
6. L. Hong, L. Y. Wang, Y. Wang, X. Wu, W. Huang, Y. Zhou, K. X. Wang, J. S. Chen, *Adv*. *Sci*. 2022, 9, 2104866.
7. M. Zhou, S. Guo, J. Li, X. Luo, Z. Liu, T. Zhang, X. Cao, M. Long, B. Lu, A. Pan, G. Fang, J. Zhou, S. Liang, *Adv*. *Mater*. **2021**, *33*, 2100187.
